# Increase of intracellular Zn^2+^ concentration directly inhibits phospholipase Cε and suppresses inflammation and tumour formation in mice

**DOI:** 10.1038/s41598-025-25886-5

**Published:** 2025-11-25

**Authors:** Yoko Yoshikawa, Motohiko Naito, Aki Emi, Kei Okada, Naoto Fujishima, Hironori Edamatsu, Shigeyuki Matsumoto, Haruka Taniguchi, Tohru Kataoka

**Affiliations:** 1 Division of Molecular Biology, Department of Biochemistry and Molecular Biology, Kobe University Graduate School of Medicine, 7-5-1 Kusunoki-cho, Chuo-ku, Kobe, 650 − 0017 Japan; 2 Divison of Molecular and Pharmaceutical Sciences, Department of Physiology and Cell Biology,, Kobe University Graduate School of Medicine, Kobe University Incubation Center Rm, 404, 1-5-6 Minatojima-Minami-machi, Chuo-ku, Kobe, 650 − 0047 Japan; 3 Present Address: Department of Drug Discovery Science, Kobe University Graduate School of Science, Technology and Innovation, 7-5-1 Kusunoki-cho, Chuo-ku, Kobe, 650 − 0017 Japan; 4 Present Address: Advanced Therapeutic Target Discovery, Department of Gastroenterology, Kobe University Graduate School of Medicine, Kobe BT Center, 1-5-6 Minatojima-Minamimachi, Chuo-ku, Kobe, 650 − 0047 Japan; 5 Present Address: Carna Biosciences, Inc., BMA 3F1-5-6 Minatojima-Minami-machi, Chuo-ku, Kobe, 650 − 0047 Japan; 6 Present Address: Juntendo University School of Medicine, 1–1 Hirakagakuendai, Inzai-shi, Chiba, 270–1695 Japan; 7 Present Address: Department of Biomedical Data Intelligence, Kyoto University Graduate School of Medicine, 53 Shogoin-Kawaharacho, Sakyo-ku, Kyoto, 606–8507 Japan

**Keywords:** PLCε, Ε class-specific PLC inhibitor, Zinc pyrithione, Zn^2+^, Anti-inflammation, Cancer prevention, Cancer, Drug discovery

## Abstract

Phospholipase Cε (PLCε) has been shown to augment inflammation and inflammation-associated carcinogenesis by inducing proinflammatory cytokine expression through activation of nuclear factor-κB (NF-κB), making it a candidate molecular target for development of anti-inflammatory and cancer-preventive agents. Aiming at developing its selective inhibitor, we carry out a high-throughput screening of 68,114 compounds by using a fluorogenic substrate and discover zinc pyrithione (ZPT) as an ε class-specific PLC inhibitor whose IC_50_ value for phosphatidylinositol 4,5-bisphosphate is 7.5 µM. Further experiments show that Zn^2+^ ion is the active principle of ZPT while the pyrithione moiety acts as an ionophore to raise the intracellular Zn^2+^ concentration. Treatment with ZPT effectively inhibits activation of protein kinase D, nuclear entry of NF-κB and expression of proinflammatory molecules induced by lysophosphatidic acid in cultured colon epithelial cells. Moreover, administration of ZPT not only ameliorates dextran sulfate-induced inflammatory colitis but also inhibits malignant progression of intestinal tumours formed in *Apc*^*Min/+*^ mice. Furthermore, it displays growth-inhibitory and anti-metastatic activities toward xenografts of human colorectal cancer cells. These results support the notion that PLCε inhibitors may become promising anti-inflammatory, cancer-preventive and anti-cancer agents, and suggest that PLCε-inhibition may account for a molecular mechanism underlying the inflammation-moderating activity of zinc.

**Supplementary Information:**

The online version contains supplementary material available at 10.1038/s41598-025-25886-5.

## Introduction

Phosphatidylinositol-specific phospholipase Cs (PLCs) catalyze the hydrolysis of phosphatidylinositol 4,5-bisphosphate (PIP_2_) into two vital second messengers, inositol 1,4,5-trisphosphate (IP_3_) and diacylglycerol (DAG). IP_3_ induces release of Ca^2+^ from the intracellular stores while DAG binds to and activates its target proteins including the isoforms of protein kinase C (PKC) and D (PKD)^1^. Thirteen PLC isoforms were identified in mammals and divided into 6 classes: β, δ, γ, ε, η and ζ^1^. PLCε is characterized by possession of two Ras-associating domains, which directly bind to small GTPases Ras and Rap, and a GEF domain, which functions as a guanine nucleotide exchange factor for Rap^2–5^. Further studies revealed its multiple regulatory mechanisms; it is regulated by not only small GTPases Ras, Rap1, Rap2B and RhoA but also heterotrimeric G protein α_12_ and β_1_γ_2_ subunits downstream of a wide variety of cell surface receptors such as those for lysophosphatidic acid and thrombin^2–7^. It is expressed in non-immune cells such as epithelial cells and fibroblasts but not in immune cells such as lymphocytes, granulocytes, macrophages and dendritic cells^8^. Analysis of the in vivo function of PLCε by applying mouse models of inflammation and carcinogenesis to *PLCε* knockout and transgenic mice demonstrated that PLCε plays pivotal roles not only in inflammation but also in carcinogenesis by augmenting cancer-associated inflammation in the microenvironment. For example, *PLCε*^*−/−*^ mice homozygous for the inactivated *PLCε* allele exhibited markedly attenuated inflammatory responses in various disease models including the dextran sulfate sodium (DSS)-induced inflammatory colitis, phorbor ester-induced skin inflammation, contact dermatitis and bronchial asthma models^8–13^, and, moreover, transgenic mice overexpressing PLCε in skin keratinocytes spontaneously developed chronic skin inflammation resembling human psoriasis^14^. Concurrently, *PLCε*^*−/−*^ mice exhibited marked resistance to tumour formation in the two-stage skin chemical carcinogenesis using 7, 12-dimethylbenz[a]anthracene as an initiator and a phorbor ester 12-*O*-tetradecanoyl-phorbor-13-acetate as a promoter as well as to the *de novo* intestinal carcinogenesis on the *Apc*^*Min/+*^ background, which were associated with attenuation of tumor-associated inflammation exemplified by reduced expression of proinflammatory molecules^9,15,16^. The role of PLCε in human carcinogenesis was supported by genome-wide association studies identifying *PLCE1* as a predisposing gene for gastric and esophageal carcinomas^17,18^. Contrary to *PLCβ*^*−/−*^ *and PLCγ*^*−/−*^ mice, which are known to display severe developmental abnormalities^1^, *PLCε*^*−/−*^ mice were born and grew normally except for the development of a mild degree of heart dilation^19^, predicting the absence of gross side effects by its pharmacological inhibition. These results introduced PLCε as a promising candidate molecular target for development of anti-inflammatory and cancer preventive agents.

Recently, we demonstrated that PLCε mediates the late phase of nuclear factor-κB (NF-κB) activation induced by tumor necrosis factor-α (TNF-α) via lysophosphatidic acid (LPA)–LPA receptor–PLCε–PKD–phosphoprotein enriched in astrocytes 15 (PEA15)–ribosomal S6 kinase (RSK)–inhibitor κB (IκB)–NF-κB pathway, thereby enhancing the NF-κB-mediated expression of proinflammatory molecules from non-immune cells^9^. In this pathway, TNF-α receptor engagement enhances the production and secretion of LPA, which induces PLCε activation in an autocrine manner. PLCε produces DAG and thereby activates PKC, leading to PKD activation through direct binding of DAG and phosphorylation by PKC. Subsequently, the activated PKD phosphorylates PEA15, a scaffolding protein, and the phosphorylated PEA15 binds to RSK, which is activated through phosphorylation by extracellular signal-regulated kinase also activated by the LPA receptor engagement. The activated RSK, held in the cytoplasm by association with the phosphorylated PEA15, phosphorylates IκB and induces its degradation by proteasome, thereby causing nuclear entry of NF-κB and activation of its transcriptional activity. Together with the early phase of activation via the canonical pathway employing IκB kinase (IKK) for IκB phosphorylation, TNF-α induces sustained activation of NF-κB.

In this study, we carry out a high-throughput screening (HTS) for PLCε inhibitors by using a fluorogenic substrate and discover zinc pyrithione (ZPT) as an ε class-specific PLC inhibitor. Further studies show that free Zn^2+^ ion is its active principle while the pyrithione moiety acts as an ionophore to raise the intracellular Zn^2+^ concentration. ZPT is also shown to display *in cellulo* and in vivo effects similar to those caused by knockout of the *PLCε* gene. Moreover, it displays growth-inhibitory and anti-metastatic effects toward cancer xenografts. These results further support the notion that PLCε inhibitors may become promising anti-inflammatory, cancer-preventive and anti-cancer agents. They also suggest that PLCε inhibition may form a molecular basis for the inflammation-moderating activity of the micronutrient zinc^20,21^, whose deficiency is known to be associated with elevated inflammation and worse outcomes in response to bacterial infection and sepsis^22^.

## Results

### Identification of ZPT as an ε-class specific PLC inhibitor

**Fig. 1:**
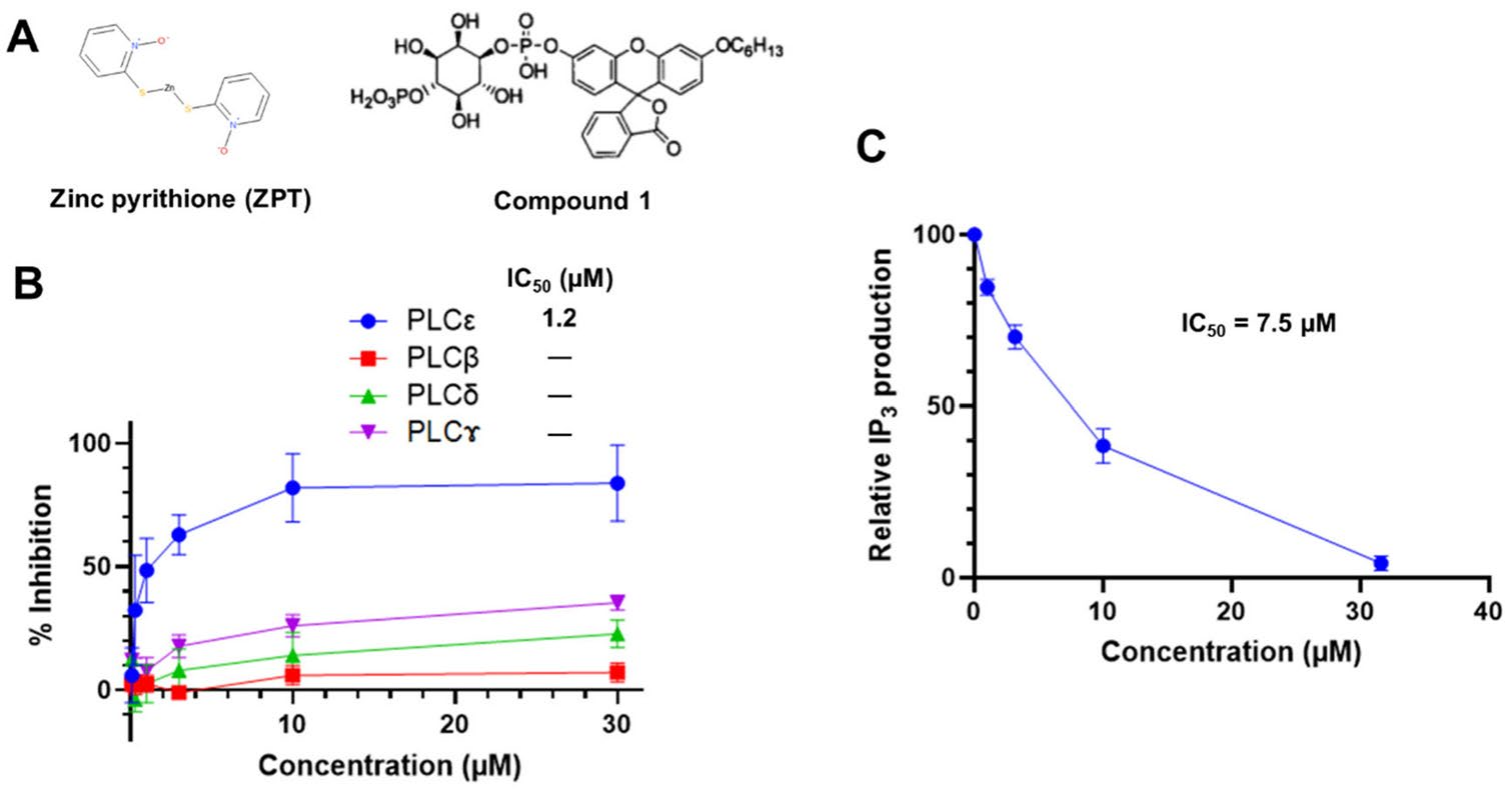
Identification of ZPT as an ε-Class Specific PLC Inhibitor. (**A**) Chemical structures of ZPT and Compound 1. (**B**) The activities of varying concentrations of ZPT to inhibit the hydrolysis of Compound 1 by purified PLCε, PLCβ4, PLCδ1 or PLCγ1 were measured as described in Methods. The experiments were performed three times in duplicates. (**C**) The activities of varying concentrations of ZPT to inhibit the hydrolysis of biotin-PIP_2_ by purified PLCε were measured as described in Methods. The experiments were performed three times.

The purified full-length PLCε polypeptide exhibited a single band with the predicted molecular size of approximately 250 kDa (Supplementary Figs. S1 and S2). A total of 68,114 compounds from Riken NPDepo Library and Riken Validated Compound Library were subjected to HTS for PLCε inhibitors utilizing this polypeptide and a fluorogenic PLC substrate Compound 1 (23, see Fig. 1A for its chemical structure) as described in Methods. The HTS identified 41 hits with the IC_50_ values of lower than 10 µM for Compound 1 (Table 1). Because ε-class selectivity of inhibition was thought to be a mandatory property considering the expected serious side effects of the inhibition of PLCγ or PLCβ^1^, all the hits were tested for the ability to inhibit the other PLC classes: β4, δ1 and γ1, which were also purified (Supplementary Figs. S1 and S2). As a result, we identified only one compound, ZPT, as a selective inhibitor of PLCε; it inhibited PLCε with the IC_50_ value of 1.2 µM but failed to noticeably inhibit PLCβ4, PLCδ1 and PLCγ1 with the IC₅₀ values of higher than 50 µM (Fig. 1A and B; Table 1 and Supplementary Fig. S3). In addition, we identified one compound displaying a certain extent of class specificity, sanguinarine, a quaternary benzo[c]phenanthridine alkaloid, with the IC_50_ values of 1.9, 80, 48 and 1.6 µM for PLCε, PLCβ4, PLCδ1 and PLCγ1, respectively (Table 1). Moreover, when a structural analogue of sanguinarine, chelerythrine (see Table 1 for its chemical structure), was purchased and examined in the same fashion, it inhibited PLCε, PLCβ4, PLCδ1 and PLCγ1 with the IC_50_ values of 1.3, 40, 7.3 and 1.8 µM, respectively. We next evaluated the inhibitory activity of ZPT toward PLCε using water-soluble biotin-linked PIP_2_ as a substrate and observed that ZPT effectively inhibited PIP_2_ hydrolysis at the IC_50_ value of 7.5 µM (Fig. 1C).

**Table 1:** Inhibitory effects of 41 compounds on PLC isoforms ε, β4, δ1 and γ1.

	Name	ε	β4	δ1	γ1		Name	ε	β4	δ1	γ1
1	6	1.2	0.7	0.5	1.3	22	53	7.9	2.4	1.2	2.1
2	21	1.4	2.4	1.4	2.4	23	67	9.0	10.1	6.1	7.2
3	11	1.6	2.3	2.7	2.4	24	63	1.3	0.9	1.6	1.3
4	Sanguinarine	1.9	79.7	47.8	1.6	25	9	2.9	3.9	3.4	4.6
5	27	2.2	2.6	2.6	1.7	26	36	3.2	0.3	2.1	0.5
6	17	2.3	2.4	1.1	2.3	27	40	3.4	3.7	3.0	3.2
7	74	2.5	2.4	1.5	2.4	28	78	3.4	2.5	2.4	2.5
8	33	2.5	2.4	1.5	2.4	29	33	3.4	3.8	3.6	4.6
9	66	2.9	2.5	2.2	1.6	30	48	3.9	4.3	2.6	4.3
10	1	3.1	3.7	0.4	8.7	31	25	3.9	3.5	3.0	4.1
11	59	3.1	2.5	2.5	2.5	32	41	4.5	2.0	1.9	3.7
12	19	3.1	2.5	1.9	2.5	33	73	4.7	3.2	35.4	1.8
13	75	3.3	2.6	1.3	2.6	34	2	4.9	4.6	5.8	1.9
14	46	3.3	4.8	2.6	9.2	35	38	5.2	5.0	3.1	6.1
15	28	3.4	3.9	3.0	4.6	36	ZPT	1.2	> 50	> 50	> 50
16	58	3.4	3.2	2.9	3.4	37	89	5.4	26.5	14.9	3.8
17	45	4.1	2.9	2.1	4.4	38	75	6.1	5.6	3.6	3.0
18	31	4.6	4.7	3.9	4.3	39	20	7.1	9.1	4.7	13.2
19	73	4.8	8.3	3.6	8.6	40	7	3.5	8.5	2.1	3.5
20	32	5.1	5.0	4.7	9.7	41	70	9.0	7.0	12.8	4.7
21	20	5.4	6.5	4.0	13.1						

### Zn^2+^ as an active principle of ZPT

**Fig. 2:**
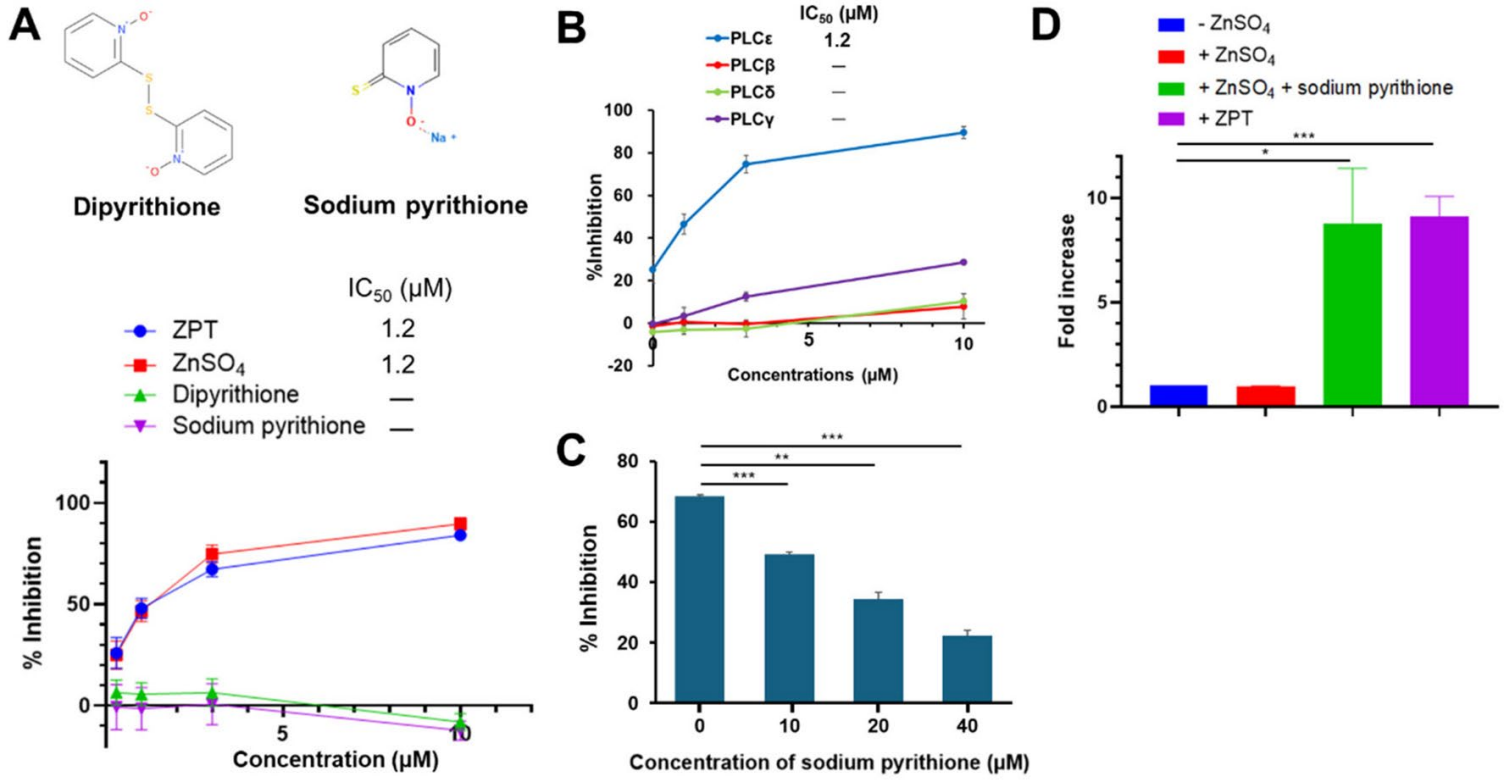
Zinc as an Active Principle of ZPT. **(A)** Chemical structures of dipyrithione and sodium pyrithione are shown. The activities of varying concentrations of ZPT, ZnSO_4_, dipyrithione and sodium pyrithione to inhibit the hydrolysis of Compound 1 by purified PLCε were measured as described in Methods. (**B**) The activities of varying concentrations of ZnSO_4_ to inhibit the hydrolysis of Compound 1 by purified PLCε, PLCβ4, PLCδ1 or PLCγ1 were measured as described in Methods. **(C)** The activities of 5 µM ZnSO_4_ to inhibit the hydrolysis of Compound 1 by purified PLCε were measured in the presence of varying concentrations of sodium pyrithione. (**D**) Caco2 cells were treated with 5 µM ZnSO_4_ in the presence or absence of 5 µM sodium pyrithione or with 5 µM ZPT for 30 min. Intracellular Zn^2+^ concentrations of the treated cells were measured by using Zinquin ethyl ester as described in Methods. The values obtained are expressed as fold changes over the average value of untreated cells. All the experiments described above were performed three times in duplicates.

In the crystalline state, ZPT exists as a centrosymmetric dimer (Fig. 1A), where Zn^2+^ forms coordination bonds with the two sulfur and two oxygen centers of the two pyrithione moieties^23^. In solution, however, the dimer is thought to dissociate via scission of one Zn-O bond. So, we next tested which of ZPT, pyrithione or free Zn^2+^ ion is responsible for the observed PLCε inhibitory activity. In vitro PLCε assay using Compound 1 as a substrate revealed that ZnSO_4_ inhibited PLCε as effectively as ZPT with the IC_50_ value of 1.2 µM (Fig. 2A) without exhibiting any noticeable inhibitory activities toward PLCβ, PLCδ and PLCγ (Fig. 2B). In contrast, sodium pyrithione and dipyrithione, where two pyrithione molecules are linked by a disulfide bond, failed to inhibit PLCε (Figs. 1A and 2A). Moreover, addition of excess sodium pyrithione to ZnSO_4_ compromised the inhibitory activity of ZnSO_4_ in a dose-dependent manner (Fig. 2C), indicating that free Zn^2+^ is the active principle of ZPT. Because pyrithione is known to possess a zinc ionophore activity that causes increased Zn^2+^ levels within mammalian cells^24–26^, we tested whether addition of pyrithione to ZnSO₄ induces an increase of intracellular Zn²⁺ level in Caco2 cells. To measure intracellular labile Zn^2+^, which comprises free and weakly protein-bound Zn^2+^, we employed a Zn²⁺-selective fluorescent indicator, Zinquin ethyl ester^27^ (Fig. 2D). While treatment with 5 µM ZnSO_4_ failed to elevate the intracellular Zn^2+^ concentration, addition of an equimolar amount of sodium pyrithione resulted in an 8.78-fold increase, confirming that pyrithione functions as a zinc ionophore. As expected, treatment with 5 µM ZPT resulted in a 9.13-fold increase in the intracellular Zn^2+^ concentration.

### Effect of ZPT on PKD–NF-κB signaling and expression of inflammation-associated genes induced by LPA

**Fig. 3:**
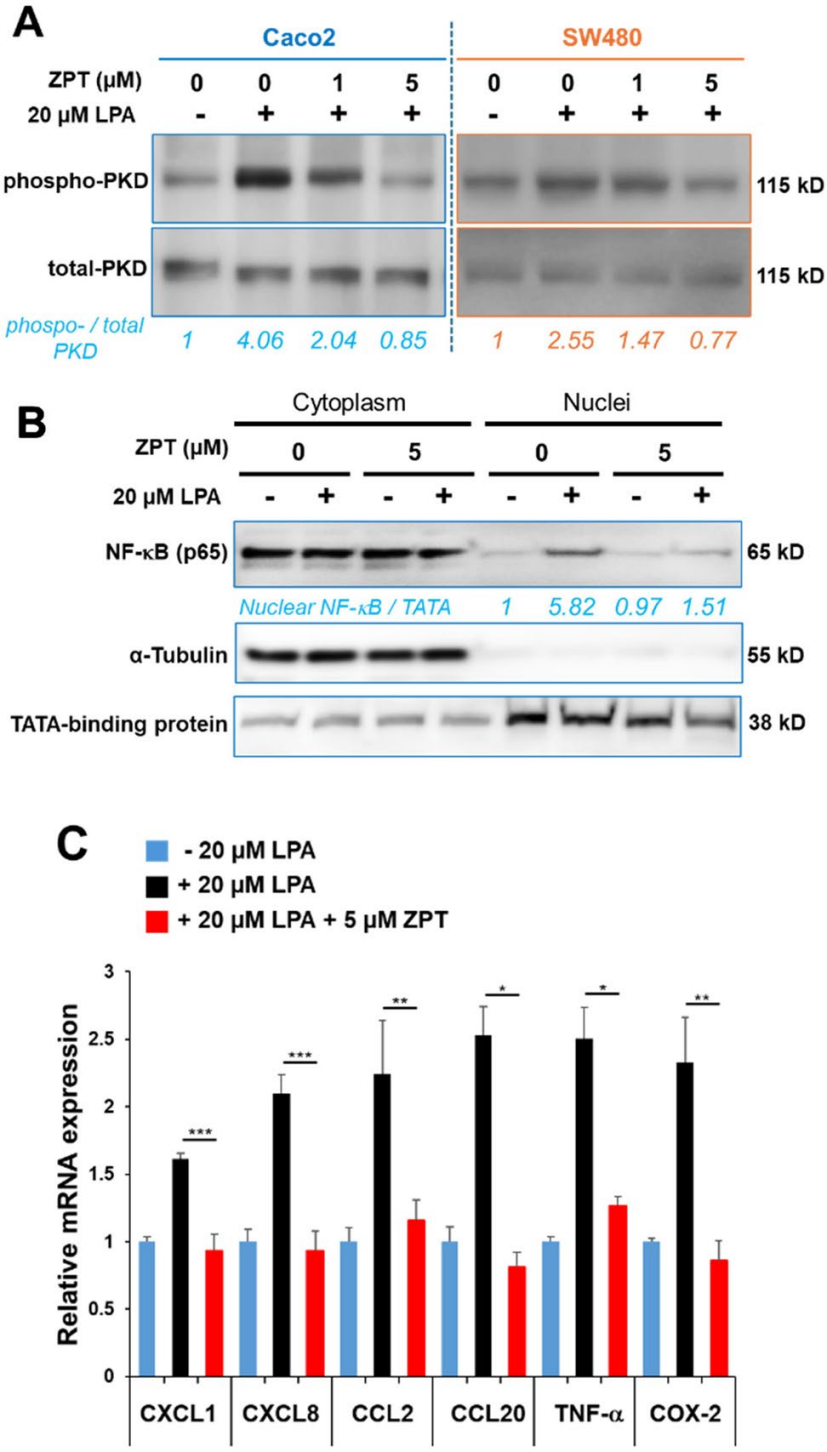
Effect of ZPT on PKD–NF-κB Signaling and Proinflammatory Gene Expression. **(A)** Caco2 and SW480 cells were serum-starved for 3 h in the presence of the indicated concentrations of ZPT or a vehicle (DMSO) and subsequently stimulated by 20 µM LPA for 30 min. PKD phosphorylated at Ser916 and total PKD in the cell lysates were quantified by immunoblotting with anti-phospho-PKD (Ser916) and anti-PKD Abs, respectively, as described in Methods. Numbers below the immunoblots indicate fold increase of the phospho-PKD signals divided by the total PKD signals over that at 0 min after LPA stimulation of the control cells. Each of the experiments was performed three times yielding equivalent results. A representative result is shown. The original blots are presented in Supplementary Figures S4 and S5. **(B)** Caco2 cells treated with 5 µM ZPT or the vehicle as described in (A) were subjected to subcellular fractionation and the resulting nuclear and cytoplasmic fractions were subjected to immunoblotting with the anti-NF-κB p65 Ab. TATA-binding protein (TBP) and α-tubulin were used as markers for the nuclear and the cytoplasmic fractions, respectively. A fold change of the nuclear NF-κB caused by 5 µM ZPT treatment is shown in numbers. Each of the experiments was performed three times yielding equivalent results. A representative result is shown. The original blots are presented in Supplementary Figure S6. **(C)** Total cellular RNAs isolated from Caco2 cells treated as described in (B) were subjected to qRT-PCR for quantification of the mRNA levels of the proinflammatory molecules: CXCL1, CXCL8, CCL2, CCL20, TNF-α and COX-2, by using the *b-*actin mRNA as an internal control. The obtained values are expressed as relative fold changes of nuclear NF-κB normalized to TATA-binding protein (TATA), with the value from the − LPA/−ZPT condition set to 1. The experiments were performed three times in duplicates.

We next examined whether ZPT possesses an *in cellulo* activity to inhibit PLCε-mediated signaling, in particular phosphorylation of PKD, nuclear translocation of NF-κB p65 and mRNA expression of proinflammatory molecules induced by LPA stimulation in Caco2 cells, where siRNA-mediated knockdown of PLCε had been shown to attenuate LPA-stimulated PLCε–PKD–PEA15–RSK–IκB–NF-κB signaling and mRNA expression of proinflammatory molecules^9^. Stimulation with 20 µM LPA led to approximately 4-fold and 2.6-fold increases in phosphorylated PKD levels in colon cancer cell lines, Caco2 and SW480, respectively. Notably, upon ZPT treatment, reduction of the phosphorylated PKD level to about a half was observed at 1 µM, and the level was reduced to almost the baseline at 5 µM in both cell lines (Fig. 3A, Supplementary Figs. S4 and S5). Moreover, treatment with 5 µM ZPT not only inhibited the nuclear translocation of NF-κB p65 (Fig. 3B and Supplementary Fig. S6) but also the induction of mRNA expression of proinflammatory molecules: CXCL1, CXCL8, CCL2, CCL20, TNF-α and COX-2, upon LPA stimulation in Caco2 cells (Fig. 3C). These results taken together with our previous study^9^ suggested that ZPT treatment inhibited the LPA–PLCε–PKD–NF-κB signaling by inhibition of PLCε through elevation of the intracellular Zn^2+^ concentration.

### Effect of ZPT administration on DSS-induced inflammatory colitis

**Fig. 4:**
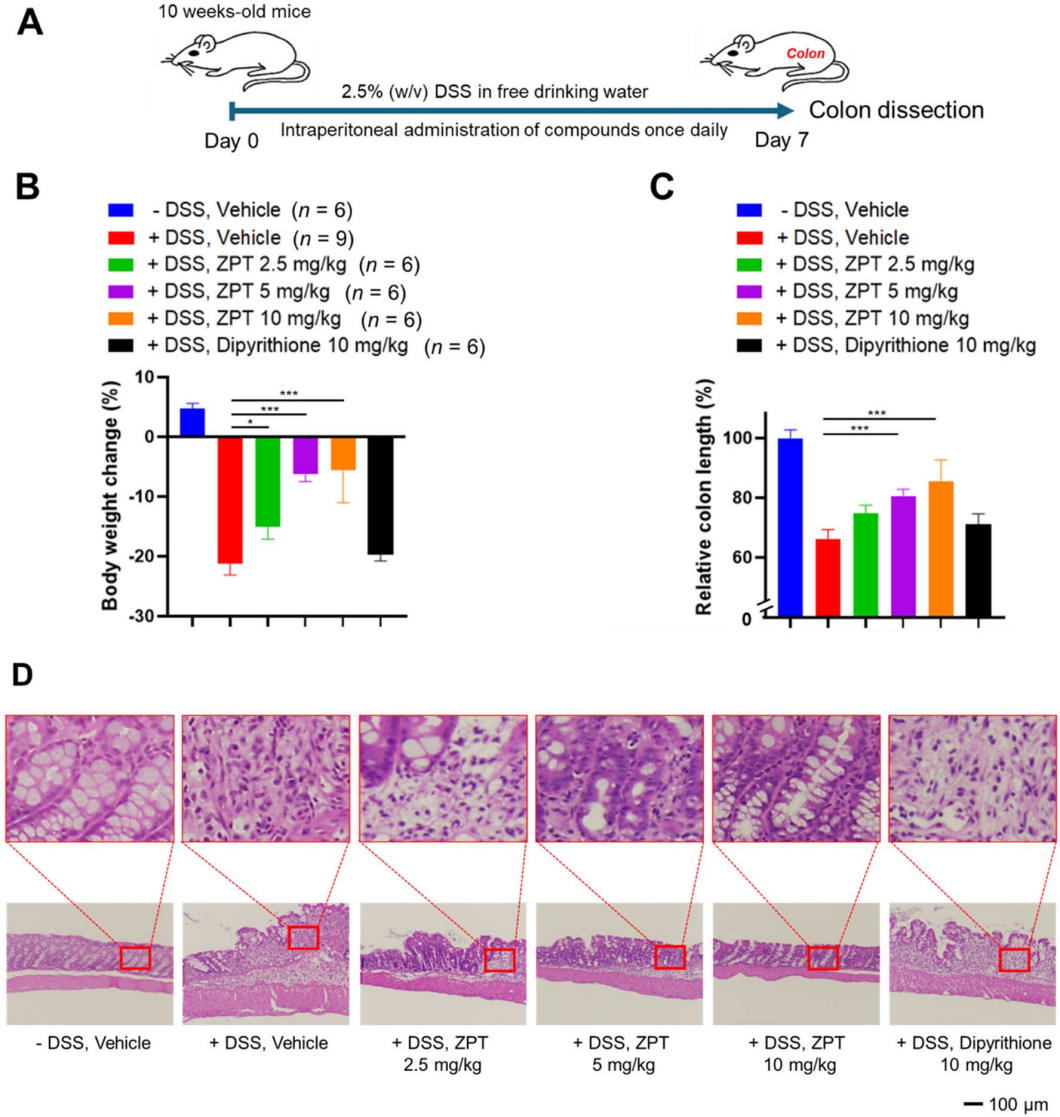
Effect of ZPT Administration on DSS-induced Inflammatory Colitis. **(A)** Mice were injected intraperitoneally with ZPT, dipyrithione or the vehicle once a day for 7 days. During this period, they were allowed to take drinking water in the presence or absence of 2.5% (w/v) DSS *ad libitum*. **(B)** Body weight changes were calculated as the body weight after 7-days treatments divided by the initial body weight. The number of mice (*n*) which received each set of the above treatments is shown in parenthesis. **(C)** Shown is the relative length of the colons dissected from the sacrificed mice after each set of the treatments compared with the untreated control mice. **(D)** Longitudinal sections of the dissected colons after each set of the treatments were subjected to H&E staining as described in Methods. A representative image of each group is shown.

We had previously shown that *PLCε*^*ΔX/ΔX*^ mice homozygous for an allele devoid of the lipase activity exhibited markedly attenuated inflammatory responses in DSS-induced inflammatory colitis, a mouse model of human inflammatory bowel diseases (IBD)^9^. Here we applied this model to evaluate the prophylactic or early intervention potential of ZPT administration. Mice were allowed to take drinking water containing 2.5% (w/v) DSS *ad libitum* for 7 days to induce acute colitis^9,28^. During this period, they received intraperitoneal injection of ZPT (2.5, 5 or 10 mg/kg), dipyrithione (10 mg/kg) or the vehicle once a day (Fig. 4A). Thereafter, the severity of colitis was evaluated by measuring the body weight loss (Fig. 4B) and the colon length shortening (Fig. 4C). DSS administration resulted in about 20% and 35% reduction in the body weight and the colon length, respectively, both of which were effectively suppressed by ZPT administration in a dose-dependent manner. Moreover, histologic examination of the H&E-stained longitudinal sections of the excised colons showed that extensive erosion of the mucosa, swelling of the submucosa and leukocyte infiltration, observed in DSS-administered mice, were effectively alleviated by ZPT administration in a dose-dependent manner (Fig. 4D). On the other hand, dipyrithione failed to alleviate any of the 3 symptoms described above.

### Effect of ZPT administration on intestinal tumourigenesis of *Apc*^*Min/+*^ mice

**Fig. 5:**
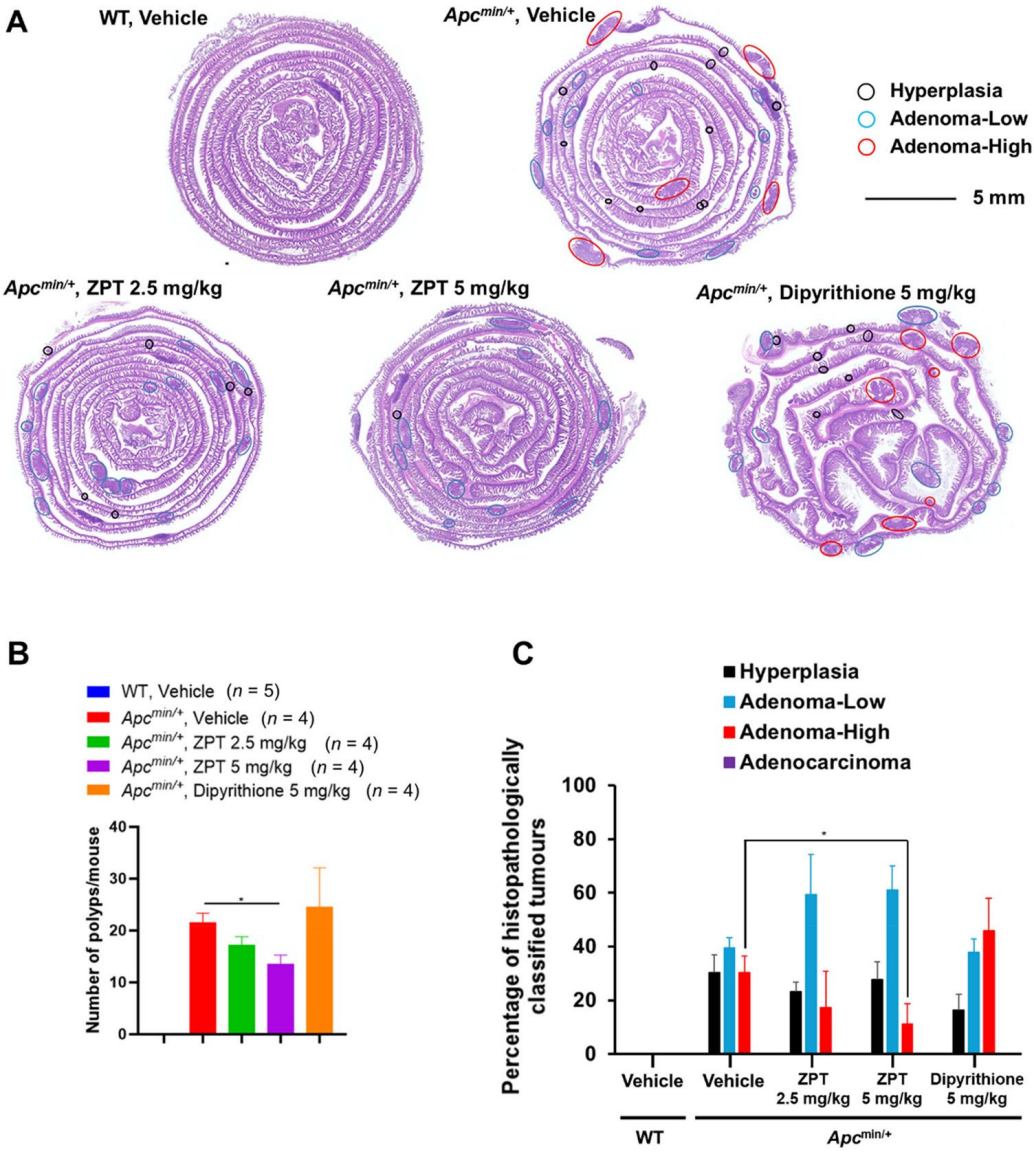
Effect of ZPT Administration on Intestinal Tumour Formation of *Apc*^*Min/+*^ Mice. *Apc*^*Min/+*^ and wild-type mice were administered intraperitoneally with ZPT, dipyrithione or the vehicle 3 days per week for 11 weeks. The number of mice (*n*) for each group is shown in Fig. B. Tumours in one longitudinal section in the middle position of the ‘Swiss roll’ of the small intestine of each mouse were subjected to H&E staining **(A)** and classified into hyperplasias, low-grade adenomas, high-grade adenomas and adenocarcinomas as described in Methods. Shown are the total numbers of the tumours per mouse **(B)** and the percentages of hyperplasias, low-grade adenomas, high-grade adenomas and adenocarcinomas **(C)**.

We next examined the effect of ZPT administration on intestinal tumour formation of *Apc*^*Min/+*^ mice, which carry a nonsense mutation in the *Apc* gene, a human *APC* ortholog, and are known to spontaneously develop multiple adenomas and a very few number of adenocarcinomas in the small intestine rather than in the large intestine^29,30^. We had previously shown that *Apc*^*Min/+*^ mice with the *PLCε*^*ΔX/ΔX*^ background exhibited marked resistance to intestinal tumorigenesis compared with those with the *PLCε*^*+/+*^ or *PLCε*^*+/ΔX*^ background, which was characterized by inhibition of malignant progression of low-grade adenomas to high-grade adenomas^9,16^. To this end, *Apc*^*Min/+*^ mice were administered intraperitoneally with ZPT (2.5 or 5 mg/kg), dipyrithione (5 mg/kg) or the vehicle 3 days per week for 11 weeks. Thereafter, one longitudinal section in the middle position of the ‘Swiss roll’ of the small intestine was subjected to H&E staining, and tumours in the whole section were histopathologically classified into hyperplasias, low-grade adenomas, high-grade adenomas and adenocarcinomas (Fig. 5A). The total numbers of tumours per mouse were reduced significantly by ZPT administration in a dose-dependent manner but not by dipyrithione administration (Fig. 5B). Without ZPT administration, the ratios of hyperplasia, low-grade adenomas, high-grade adenomas and adenocarcinomas were approximately 30, 40, 30, and 0%, respectively (Fig. 5A and C). ZPT administration resulted in a marked change; the ratio of high-grade adenomas substantially decreased (Fig. 5C). These results showed that the progression of low-grade adenomas to high-grade adenomas was suppressed by ZPT administration as observed in *PLCε*^*ΔX/ΔX*^ mice^9,16^, implying that PLCe played a facilitative role in this stage of tumour promotion. Unexpectedly, treatment with dipyrithione appeared to accelerate the progression of low-grade adenomas to high-grade adenomas, for which we do not understand the reason at present.

### Effect of ZPT administration on growth of tumour xenograft

**Fig. 6:**
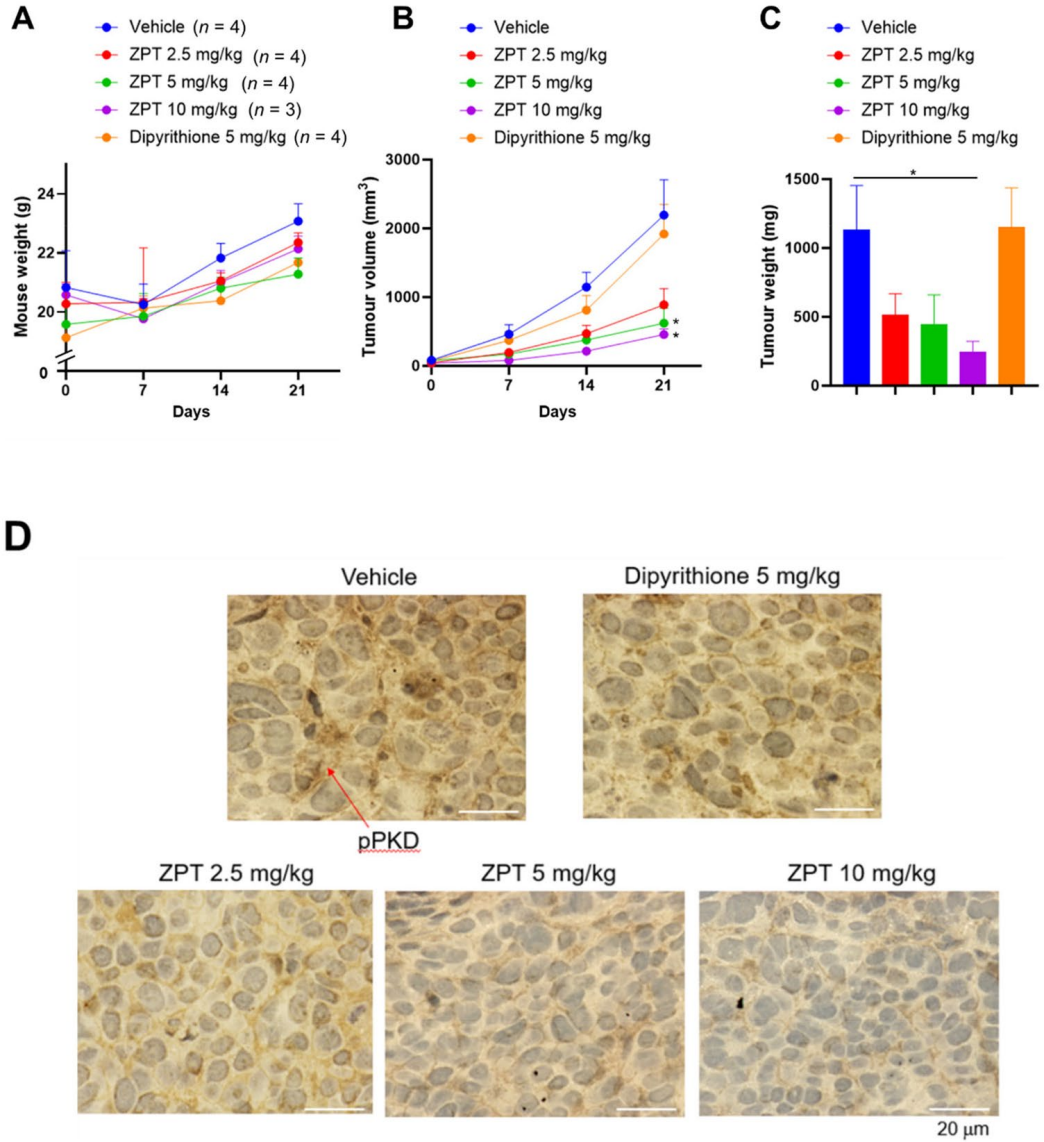
Effect of ZPT Administration on Proliferation of Xenografted SW480 cells. SW480 cells (5 × 10^6^ cells) were implanted subcutaneously into the right flanks of female nude mice. One week later, the mice were administered intraperitoneally with ZPT, dipyrithione or the vehicle 3 days per week for 3 weeks, during which the body weight **(A)** and the tumour volumes **(B)** were measured every 7 days. The number of mice (*n*) for each group is shown in parenthesis. Thereafter, the tumours were excised and weighed **(C)**, and their sections were subjected to immunohistochemical detection of phosphorylated PKD by using anti-phospho-PKD antibody **(D)**.

**Fig. 7:**
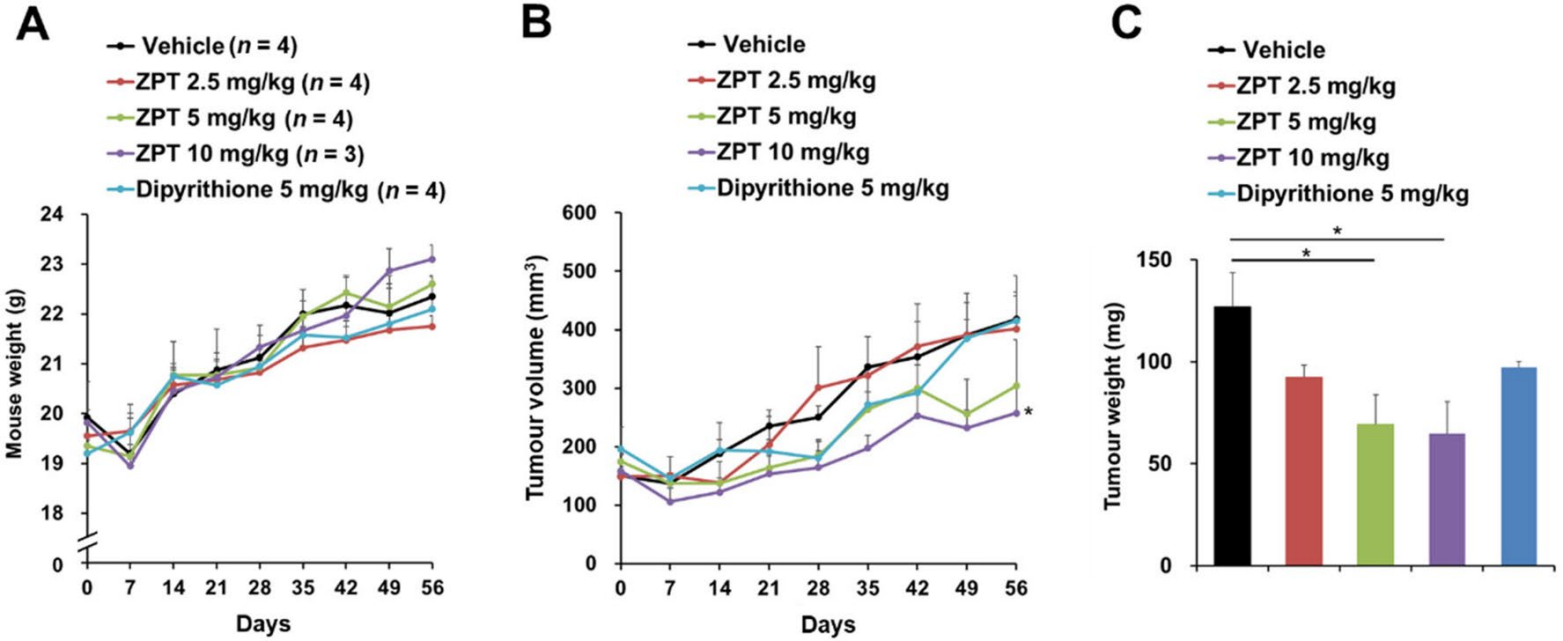
Effect of ZPT Administration on Proliferation of Xenografted Caco2 cells. Caco2 cells (5 × 10^6^ cells) were implanted subcutaneously into the right flanks of female nude mice. One week later, the mice were administered intraperitoneally with ZPT, dipyrithione or the vehicle 3 days per week for 8 weeks, during which the body weight **(A)** and the tumour volumes **(B)** were measured every 7 days. The number of mice (*n*) for each group is shown in parenthesis.　The number of mice (*n*) for each group is shown in parenthesis. Thereafter, the tumours were excised and weighed **(C)**.

Since it was well established that proliferation and metastasis of cancers are profoundly influenced by their inflammatory microenvironment, we examined the effect of ZPT administration on growth of human colon cancer cells xenografted on nude mice. One week after subcutaneous implantation of SW480 or Caco2 cells into their right flanks, female nude mice were administered intraperitoneally with ZPT (2.5, 5 or 10 mg/kg), dipyrithione (5 mg/kg) or the vehicle 3 days per week for 3 and 8 weeks, respectively, during which the body weight (Figs. 6A and 7A) and the tumour volumes (Figs. 6B and 7B) were monitored every 7 days. The administration of ZPT but not dipyrithione effectively inhibited the growth of both of the grafted SW480 and Caco2 cells in a dose-dependent manner without significant loss of the body weight. Also, it resulted in a marked decrease in the weight of the isolated tumours at the final day of the experiment (Figs. 6C and 7C). Immunohistochemical analysis of the tumours in the SW480-grafted mice revealed that the cellular level of phosphorylated PKD was markedly reduced by ZPT administration in a dose-dependent manner, supporting the inhibition of the PLCε-mediated signaling (Fig. 6D). These results indicated that ZPT possesses growth-inhibitory activity toward the two different colon cancer xenografts.

### Effect of ZPT administration on lung metastasis of tumour xenograft

**Fig. 8:**
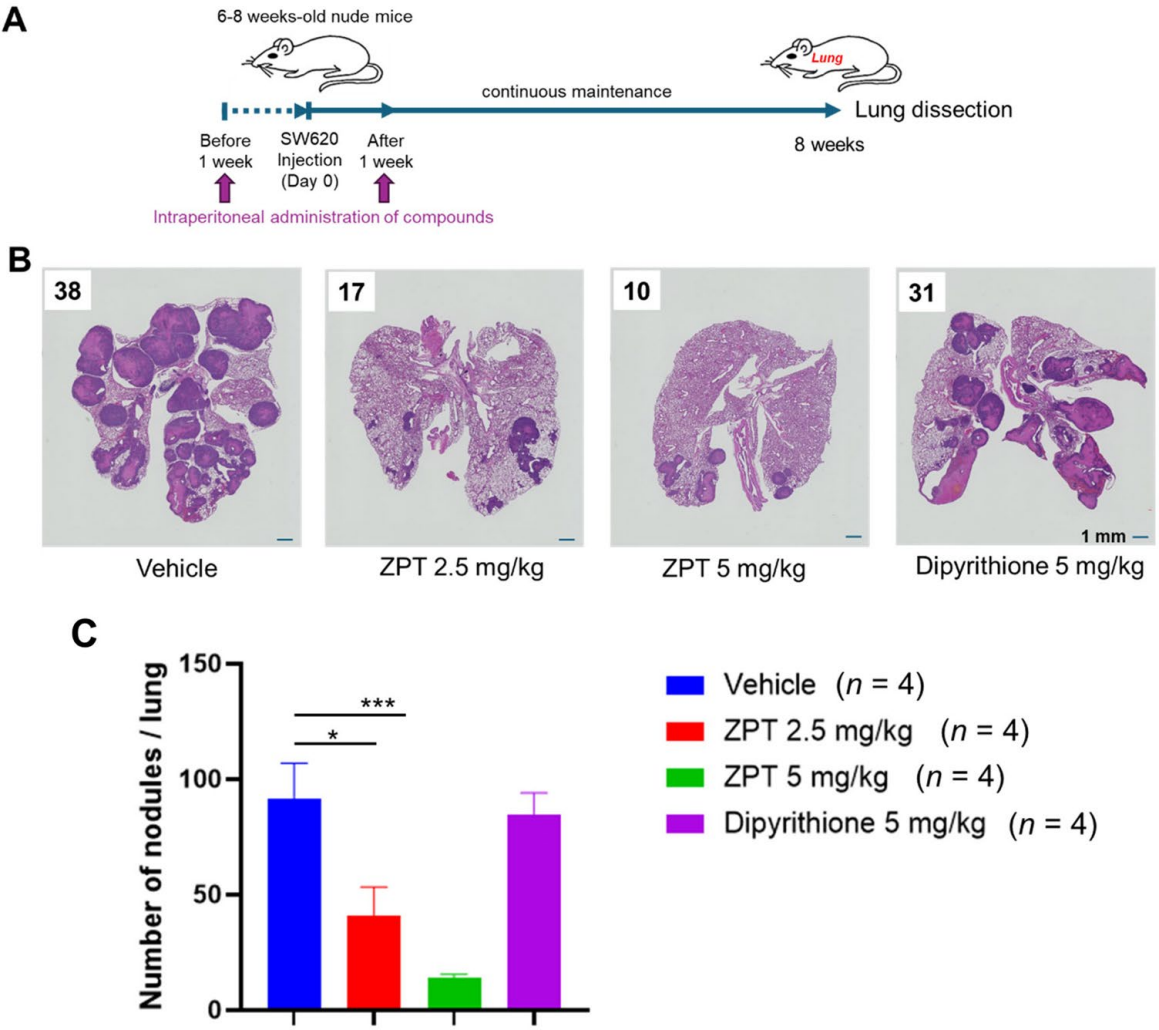
Effect of ZPT Administration on Lung Metastasis of Tumour Xenograft. **(A)** Female nude mice were injected with SW620 cells (1 × 10^7^ cells) via tail vein and maintained for further 8 weeks. ZPT, dipyrithione or the vehicle was administered intraperitoneally to the mice twice, one week before and after the cell injection (*n* = 4 for each group). Thereafter, the lungs were excised and their sections were subjected to H&E staining for histologic evaluation of lung micro-metastasis under microscope as described in Methods. **(B)** Shown is a representative image of the H&E-stained section of each group, where the locations of hyperplasias, low-grade adenomas and high-grade adenomas are encircled by indicated colours. Also, the numbers of micro-metastatic nodules counted under a light microscope are shown. **(C)** The numbers of micro-metastatic nodules in 3 non-contiguous sections of each mouse are shown.

Female nude mice were injected with SW620 cells via tail vein and maintained for further 8 weeks (Fig. 8A). ZPT (2.5 or 5 mg/kg), dipyrithione (5 mg/kg) or the vehicle was administered intraperitoneally to the mice twice, one week before and after the cell injection. Shown are a representative image of the H&E-stained lung section of each group (Fig. 8B) and the total number of micro-metastatic nodules in 3 non-contiguous lung sections from each mouse (Fig. 8C). The results clearly showed that the numbers of the micro-metastatic modules were effectively reduced by ZPT administration in a dose-dependent manner whereas dipyrithione administration exhibited no effect. These results indicated that ZPT possesses an anti-metastatic activity toward the tumour xenograft.

### Expression of PLCε in human cancers

Because there was an argument questioning the tumour-promoter role of PLCe based on the observation that its expression was significantly downregulated in clinical samples and cell lines of some cancers, in particular colorectal and lung cancers^31^, we systematically evaluated the expression status of PLCε in various types of cancers using public databases. At first, we analyzed transcriptomic data on *PLCE1* from The Cancer Genome Atlas (TCGA) using the GEPIA2 platforms^32,33^, where *PLCE1* mRNA levels were measured in a large number of tumour samples belonging to various cancer types and matching normal tissues and their average values were determined (Supplementary Fig. S7). The expression levels of *PLCE1* showed large variation among tumour samples belonging to the same cancer types and exhibited low cancer-type specificity. Statistical analysis revealed no significant differences in the average *PLCE1* expression levels between tumour samples and normal tissues in all the cancer types including adenocarcinomas of colon, rectum, stomach, esophagus and lung. Next, we analyzed *PLCE1* mRNA levels in various colorectal cancer cell lines from the Human Protein Atlas (HPA) database^34^ (Supplementary Fig. S8). The result indicated that *PLCE1* expression levels of all the cell lines including Caco-2, SW480 and SW620, used in the present study, were comparable to the average levels of normal colon tissues from TCGA.

## Discussion

Past studies on PLCε using genetically modified mice had strongly suggested that PLCε might be a promising candidate molecular target for development of anti-inflammatory and cancer preventive agents without any gross side effects, leading us to develop its selective inhibitors in this study. There had been little progress in the development of PLC inhibitors due to the difficulty in HTS, which was mainly ascribable to the water-insoluble nature of PIP_2_. Although this problem had been overcome by the development of water-soluble and fluorogenic PLC substrates^35–37^, PLC inhibitors with high potency, especially those having any class selectivity, remained to be identified. Although U73122 had been widely used as a pan-PLC inhibitor at the cellular level, it failed to inhibit PLC catalytic activity in vitro and molecular mechanism of its action remained unclear^37^. In this study, we carried out an HTS for PLCε inhibitors using the fluorogenic substrate Compound 1 and discovered ZPT as a potent ε class-specific PLC inhibitor with the IC_50_ value of 7.5 µM using PIP_2_ as a substrate (Fig. 1). The ε-class selectivity was a mandatory property for therapeutic application considering the expected serious side effects of the inhibition of PLCγ and PLCβ^1^. Further studies showed that free Zn^2+^ ion was responsible for the PLCε inhibitory activity of ZPT while the pyrithione moiety acted as an ionophore to raise the intracellular Zn^2+^ concentration (Fig. 2). While the total cellular Zn^2+^ concentration is thought to range between tens and hundreds of micromolar^21,38^, the vast majority is bound to hundreds of proteins with diverse affinity or sequestered into organelles and vesicles, so that the cytosolic free Zn^2+^ concentration is less than low nanomolar^39,40^. Moreover, the cellular and subcellular distribution of zinc is under sophisticated regulation by a number of zinc transporters, ZnTs and ZIPs^21^. Thus, strictly speaking, we were not fully certain whether the intracellular Zn^2+^ level achieved by ZPT treatment was high enough to directly inhibit PLCε in the presence of the physiological concentration of PIP_2_ although 9.13-fold increase of labile Zn^2+^ was observed by using Zinquin. However, our observation of the cellular effects of ZPT treatment (*vide infra*) strongly supported that the intracellular Zn^2+^ level achieved was indeed high enough to inhibit PLCε.

ZPT treatment showed cellular effects similar to those caused by siRNA-mediated knockdown of PLCε in colon epithelial Caco2 cells^9^: namely, inhibition of the LPA-induced PKD phosphorylation and NF-κB nuclear translocation as well as inhibition of the LPA-induced expression of the proinflammatory molecules: CXCL1, CXCL8, CCL2, CCL20, TNF-α and COX-2, which were known to be regulated by NF-κB^41^ (Fig. 3). It also inhibited the LPA-induced PKD phosphorylation in another colon cancer SW480 cells. This led us to examine the in vivo effects of ZPT administration by employing mouse models of inflammation and carcinogenesis, which had been used by us for characterization of *PLCε* knockout mice. Although the ameliorating effect of ZPT administration on DSS-induced inflammatory colitis had already been reported before^42^, we used this model to compare the effect of ZPT with that of *PLCε* knockout on 3 symptoms^9^ and observed no significant difference (Fig. 4). In this regard, of particular interest was the role of proinflammatory chemokines: CXCL1 and CXCL8 (IL-8), which recruit neutrophils through engagement with their receptor CXCR2, and CCL2, which recruits monocytes and macrophages through engagement with its receptor CCR2, because it had been shown that mice deficient in CXCR2 or CCR2 became less susceptible to DSS-induced colitis^43–47^ and that CXCL8, which is non-existent in mouse, played a crucial role in pathogenesis of human IBDs^43,48–50^. These findings taken together suggested a mechanism whereby PLCε enhances the production of CXCL1, CXCL2 (a CXCR2 ligand), CXCL8 and CCL2 from the colon epithelial cells, thereby recruiting neutrophils, monocytes and macrophages to accelerate colon inflammation. This was supported by our previous observation that CXCL1, CXCL2, CCL2 and CXCR2 were overexpressed in the colon epithelial cells of the DSS-administered *PLCε*^+/+^ mice, which was substantially attenuated in those of the DSS-administered *PLCε*^*ΔX/ΔX*^ mice^16^. Although not tested in this study, other CXCR2 ligands: CXCL3, 5, 6 and 7, and other CCR2 ligands: CCL7, 8, 12 and 13, may also play a critical role. Moreover, CCL20, TNF-α and COX-2 were likely to play facilitative roles because they had been implicated in the pathogenesis of DSS-induced colitis and human IBDs^43,51–55^. In particular, TNF-α was expected to exert an amplifying effect on proinflammatory responses through induction of NF-κB activation.

ZPT administration showed an inhibitory effect on the progression of low-grade adenomas to high-grade adenomas formed in *Apc*^*Min*^ mice, a widely used animal model for human colorectal carcinogenesis, where tumour environment, in particular inflammation, was known to play a critical role in tumour promotion^56,57^ (Fig. 5). This result was very similar to that we had previously observed in *PLCε*^*ΔX/ΔX*^ mice with the *Apc*^*Min/+*^ background^9,16^ and further supported our notion that PLCe plays a facilitative role in the stage of transition from low-grade to high-grade adenomas. The results appeared to be compatible with our previous observation by immunohistochemistry with anti-PLCe antibody that PLCε was expressed in low-grade adenoma cells frequently colocalizing with VEGF while its expression was downregulated in high-grade adenoma cells^16^. High-grade adenomas of *PLCε*^*ΔX/ΔX*^;*Apc*^*Min/+*^ mice exhibited marked attenuation of tumour-associated inflammation^16^, in which the attenuation of COX-2 expression was likely to play a critical role because pharmacological inhibition or genetic ablation of COX-2 had been shown to suppress the progression of colorectal tumours^57,58^. This was consistent with the past observation that the main effect of inflammation on tumourigenesis is exerted in the late stage, where tumours progress to high-grade adenomas^56,59^. Thus, we obtained further support for our hypothesis that the proinflammatory function of PLCε is responsible for its crucial role in facilitating malignant progression. However, it must be noted that a fundamental controversy exists over the role of PLCε in carcinogenesis^31^. Experiments applying the two-stage skin chemical carcinogenesis model to different types of *PLCε* knockout mice yielded two opposing results; our group observed a tumour-promoter role by employing *PLCε*^*ΔX/ΔX*^ mice^15^ while Martins et al.. observed a tumour-suppressor role by employing mice carrying deletion of the whole *PLCε* gene^60^. In this line, it may be worth noting that Berghe et al.. reported that the epidermal-thickening phenotype observed in the *PLCe* knockout mice of Martins et al.. was possibly ascribed to a passenger mutation of an adjacent gene *AOX4*, encoding aldehyde oxidase 4, derived from 129 strain used for gene targeting^61^. This raises a possibility that the increased incidence of skin cancer observed by Martins et al.. might also be affected by the *AOX4* passenger mutation. Also, the argument against the tumour-promoter role of PLCε based on its expression levels seems to lack sufficient evidence as described in Results. Although further investigation is needed to solve the controversy, our present results using the selective PLCe inhibitor ZPT would provide a further support for the tumour-promoter role of PLCe.

Furthermore, we found that ZPT administration showed inhibitory effects on growth and metastasis of human cancer cells xenografted on nude mice (Figs. 6, 7 and 8). It was well established that proliferation and metastasis of cancer cells are profoundly influenced by the surrounding microenvironment (tumour stroma) consisting of stromal cells, such as cancer-associated fibroblasts, mesenchymal stem cells, endothelial cells, pericytes, tumor-associated macrophages (TAMs) and tumour-associated neutrophils (TANs), which are embedded in the extracellular matrix^62–64^. Various stromal cells interact with each other or with epithelial cancer cells and produce numerous bioactive factors inducing proliferation, survival, angiogenesis, invasion and metastasis, epithelial-mesenchymal transition etc., of tumours. For example, TAMs and TANs are recruited to the tumour stroma by the action of chemokines secreted from cancer cells, such as CXCL1, CXCL2, CXCL8 and CCL2, and become a secondary source of chemokines which affect tumour proliferation, angiogenesis and metastasis, such as CXCL1, CXCL2, CXCL8 and CCL2^64,65^. Although it is possible that inhibition of the expression of CXCL1, CXCL2, CXCL8 and CCL2 might at least in part account for the growth-inhibitory and anti-metastatic effects of ZPT administration, we do not have any additional data hinting the underlying molecular mechanism.

Zinc, an essential trace element, facilitates the coordination of innate and adaptive immunity^20,21^. Zinc deficiency causes immune dysfunction resulting in increased morbidity and mortality following infection^22^, whereas zinc supplementation prevents the incidence of infectious diseases and improves immune function^66,67^. Past studies suggested that zinc might negatively regulate NF-κB activity during innate immune activation^68,69^. As for the underlying mechanism, it was reported that the activity of IKKβ is directly inhibited by Zn^2+^, whose intracellular level is increased through up-regulation of the zinc transporter SLC39A8 (ZIP8) by NF-κB in lung epithelial cells, monocytes and macrophages^70^. In mast cells, loss of the zinc exporter Slc30a5 (Znt5) resulted in increased labile Zn^2+^ and suppressed NF-κB signaling in response to FcεR1 stimulators^71^. Thus, it is likely that, in epithelial cells, the ZPT-induced increase of the intracellular Zn^2+^ concentration inhibits NF-κB activation through dual inhibition of IKKβ and PLCε, thereby negatively regulating proinflammatory responses. It may be noteworthy to point out that chelerythrine and sanguinarine, which were shown to inhibit PLCε as potently as ZPT and Zn^2+^ in this study, have been used in folk medicine^72^ for their wide range of biological effects including not only anti-inflammatory activity mediated by downregulation of NF-κB and proinflammatory cytokine expression but also anti-tumour activity^72–74^, hinting that PLCε inhibition might form a molecular basis for their action.

Our results using ZPT as a model compound suggested that selective PLCε inhibitors might become promising anti-inflammatory, cancer-preventive and anti-cancer agents with a novel mechanism of action. The anti-cancer effect is based on both growth-inhibitory and anti-metastatic activities. They are expected to have a broad range of applications in view of the attenuating effects of PLCε deficiency toward diverse sets of inflammation and carcinogenesis^8–13,15,16^. Of particular interest is the cancer prevention, which has tended to be neglected as a drug target by pharmaceutical companies mainly due to difficulties in clinical trials regarding the issues of long-term administration and accompanying chronic side effects. ZPT is an antifungal and antibacterial agent and has been used in the treatment of seborrheic dermatitis and dandruff of the scalp as an ingradient of shampoos and cosmetics since the 1960s^75^. However, its use was recently prohibited in some countries because of the potential reproductive and environmental toxicity^76^. Also, ZPT exhibits acute, repeated-dose and chronic toxicity in the oral, dermal and inhalation routes^77^. Accordingly, ZPT, as such, seems to be unsuitable for medical use and therefore the development of selective PLCε inhibitors with low toxicity is needed for their clinical application as anti-inflammatory, cancer preventive and anti-cancer agents.

## Methods

### Antibodies and chemicals

Following antibodies were commercially obtained: rabbit anti-PKD Ab (#2052, Cell Signaling Technologies), rabbit anti-DYKDDDDK Tag (D6W5B) Ab (#14793, Cell Signaling Technologies), rabbit anti-phospho-PKD (Ser916) Ab (#2051, Cell Signaling Technologies), rabbit anti-p65/RelA Ab (sc-8008, Santa Cruz), anti-α-tubulin Ab (#3873, Cell Signaling Technologies), rabbit anti-TATA-binding protein (TBP) Ab (sc-273, Santa Cruz) and rabbit anti-FLAG-tag Ab (PM020, MBL). Following chemicals were commercially obtained: ZPT [bis(2-pyridylthio)zinc 1,1’-dioxide], dipyrithione and sodium pyrithione (Sigma), MG-132 ((proteasome inhibitor, Calbiochem), 1-oleoyl-2-hydroxy-sn-glycero-3-phosphate (LPA) (Avanti), ZnSO_4_ and ZnCl_2_ (Nacai tesque, Kyoto, Japan), sanguinarine (Toronto Research Chemicals, Toronto, Canada) and chelerythrine chloride (Adipogen Life Sciences). A fluorogenic PLC substrate, [(6’-hexoxy-3-oxo-spiro[isobenzofuran-1,9’-xanthene]−3’-yl)oxy-[(2R,3R,5 S,6R)−2,3,4,5,6-pentahydroxycyclohexoxy]phophoryl]oxylithium (Compound 1)^35^ was custom-synthesized by Sapala Organics Pvt. Ltd. (Hyderabad, India). They were used according to the manufacturers’ recommendations.

### Cell lines

A human colon cancer epithelial cell line Caco2 was purchased from ATCC (HTB-37) and maintained in 5% CO_2_ at 37 °C in modified Eagle’s minimum essential medium (MEM) (Nacalai tesque) supplemented with 20% fetal bovine serum (FBS) (Sigma), non-essential amino acids (Gibco) and 100 µg/ml penicillin-streptomycin (Nacalai tesque). Human colorectal cancer cell line SW480 harboring the *K-ras*^G12V^ gene and its metastatic subclone SW620 were obtained from the European Collection of Cell Cultures and cultured in L-15 medium (Gibco) containing 10% FBS.

### Animals

Animals were housed under the temperature (22 ± 1 °C) and humidity (45–65%)-controlled environment with a 12-h light-dark cycle. *Apc*^*Min/+*^ mice were obtained from the Jackson laboratory (Bar Harbor, ME). Female athymic nude (nu/nu) mice, C57BL/6JJc1 mice and B6 mice were obtained from CLEA Japan, Tokyo, Japan. All the animals were maintained in the animal facility of Kobe University Graduate School of Medicine and the use and care of the animals were reviewed and approved by the Institutional Animal Care and Use Committee of Kobe University. All methods were carried out in accordance with relevant guidelines, regulations, and ARRIVE guidelines. Euthanasia for the purpose of providing mouse tissue was performed with CO_2_ exposure followed by cervical dislocation.

### Purification of full-length PLC polypeptides

Exponentially growing Expi293F cells (4 ~ 5 × 10^6^ cells) in 25 ml of Expi293 Medium (Gibco) were transfected with 30 µg each of pFLAG-CMV2 plasmids (Sigma) expressing the full-length polypeptides of human PLCε, PLCβ4, PLCδ1 and PLCγ1 as FLAG fusions^9^ by using ExpiFectamine 293 Transfection Kit and Opti-MEM^®^1 (Gibco), and incubated for 48 h in a 125-ml Erlenmeyer flask with constant rotation in a 37 °C incubator with 80% relative humidity and 8% CO_2_. After incubation, the cells were collected, lysed by sonication in a lysis buffer {50 mM Tris-HCl, pH 7.4, 150 mM NaCl, 1 mM MgCl_2_, 1 mM EDTA, 1 mM dithiothreitol (DTT), 10% glycerol and 1% Triton X-100} and centrifuged at 35,000 rpm for 30 min at 4 °C. FLAG-tagged PLC polypeptides in the resulting supernatant were immobilized on 200 µl slurry of DDDDK-tagged Protein PURIFICATION GEL (MBL) and eluted 5 times with 100 µl each of DDDDK-tag peptide elution buffer {0.5% sodium cholate, 1 mM DTT and 0.1 mg/ml DDDDK-tag peptide (MBL)} in phosphate-buffered saline (PBS). The eluted proteins were separated by SDS-polyacrylamide gel electrophoresis (SDS-PAGE), blotted with rabbit anti-DYKDDDDK Tag (D6W5B) Ab for quality check and rough quantification.

### In vitro PLC assay using a fluorogenic substrate and HTS for PLCε inhibitors

Ten µl of 2 × substrate solutions (50 mM HEPES pH7.2, 200 mM NaCl, 0.2 mM CaCl_2_, 0.4 mM DTT, 0.4 µM EDTA, 0.02% gelatin, 0.2% sodium cholate and 60 µM Compound 1) were added to each well of a 384-well plate (CONING #3573) containing 10 µl of purified PLCε (120 ng) and incubated with shaking at 30 °C for 3 h. After terminating the reaction by the addition of 2 µl of 5 mM EDTA, the fluorescence emission was measured at 520 nm with the excitation at 485 nm by a microplate reader (POLARstar Omega-25). For measuring inhibition by compounds for HTS, the reactions were conducted at RIKEN Center for Sustainable Resource Science (CSRS) as described above except that 5 µl of compound solution and 5 µl of purified PLCe (120 ng) were added to each well. Solution dispensing and fluorescence detection were conducted by using an automated dispenser (EDR-384, Biotec) and a hybrid multi-mode microplate reader (Synergy H4, Biotec), respectively. For determination of the IC_50_ values for ZPT and Zn^2+^, the EDTA concentration was reduced to one-tenth to avoid its zinc-chelating effect. For assay of the other PLC classes, the reactions were performed similarly except that PLCβ4 (5 ng), PLCδ1 (0.1 ng) or PLCγ1 (0.3 ng) was used instead of PLCε.

### In vitro PLC assay using PIP_2_ as a substrate

Purified PLCε (5.4 µg) was incubated with 0.6 µg of water-soluble biotin-PIP_2_ (Echelon Biosciences) in 60 µl of a reaction buffer (25 mM HEPES pH7.2, 100 mM NaCl, 0.1 mM CaCl_2_, 0.1 mM DTT, 0.01% gelatin and 0.1% sodium cholate) at 30 ℃ for 3 h in the presence of varying concentrations of ZPT. After the reaction, a 50-µl aliquot was subjected to the measurement of IP_3_ using the human IP_3_ ELISA kit (Cusabio) according to the manufacturer’s protocol.

### Determination of intracellular zinc concentration

Confluent Caco2 cells in a Φ10-cm culture dish were subjected to serum starvation overnight and incubated with 10 µM MG132 for 30 min. After the addition of 5 µM ZnSO_4_ in the presence or absence of 5 µM sodium pyrithione, 5 µM ZPT or the vehicle, the cells were stimulated by 20 µM LPA for 30 min. The cells were harvested by trypsinization, washed with PBS and suspended at the density of 5 × 10^6^ cells/ml in Hanks’ balanced salt solution (HBSS). After the addition of 1 mg/ml Zinquin ethyl ester (Dojindo Laboratories, Kumamoto, Japan)^27^ dissolved in DMSO at the final concentration of 25 µM, the cell suspension was incubated at 37 °C for 30 min. Subsequently, the cells were washed three times with HBSS, suspended in HBSS at the density of 5 × 10^6^ cells/ml and subjected to measurement of the fluorescence emission at 490 nm with the excitation at 370 nm by POLARstar Omega-25.

### LPA-Induced PKD phosphorylation and proinflammatory gene expression

Confluent Caco2 and SW480 cells were serum-starved for 3 h in the presence of ZPT or a vehicle (DMSO) and stimulated by 20 µM LPA for 30 min. The cells were lysed in a lysis buffer {20 mM Tris-HCl, pH7.4, 250 mM NaCl, 3 mM EDTA, 0.5% (v/v) Nonidet P-40 and protease inhibitor cocktail (Nacalai tesque)}. The lysates were centrifuged at 21,500 × *g* for 10 min and the supernatants were used for immunoblotting with anti-PKD and anti-phospho-PKD (Ser916) Abs, followed by quantification of the immunoreactive signals using ImageQuant LAS4000mini and ImageQuant TL (GE Healthcare) as described^9^. Total cellular RNA isolation from the LPA-stimulated Caco2 cells, cDNA synthesis, reverse transcription-polymerase chain reaction (RT-PCR) and quantitative (q) RT-PCR were performed as described previously^9^. Relative mRNA level of each transcript was determined by the ΔΔCt method with b-actin as a reference gene. The primers used are listed in Supplementary Table 1.

### Subcellular fractionation

Subcellular fractionation was conducted as described^9^. Briefly, the LPA-stimulated Caco2 cells were harvested using a scraper in ice-cold PBS and, after centrifugation, the cell pellet was resuspended in Lysis buffer A {10 mM Hepes, pH 7.9, 10 mM KCl, 1.5 mM MgCl_2_, 0.1 mM EDTA, 0.25% (v/v) Nonidet P-40 and protease inhibitor cocktail}. After centrifugation at 1,000 × *g* for 10 min, the supernatant was used as the nuclear fraction. The pellet was resuspended in a nuclear extraction buffer {20 mM Hepes, pH 7.9, 420 mM NaCl, 1.5 mM MgCl_2_, 0.1 mM EDTA, 1 mM DTT, 25% (v/v) glycerol and protease inhibitor cocktail} and incubated for 30 min. After centrifugation at 21,500 × *g* for 10 min, the supernatant was used as the cytoplasmic fraction.

### Induction of colitis and histologic analyses

To induce inflammatory colitis, 10 weeks-old C57BL/6JJc1 mice were allowed to take drinking water containing 2.5% (w/v) DSS (molecular weight 36,000 ~ 50,000; Wako Pure Chemical, Osaka, Japan) *ad libitum* for 7 days^9,30^. During this period, they were administered intraperitoneally with ZPT, dipyrithione or a vehicle (0.5% carboxymethyl cellulose and 0.9% NaCl) once a day. Thereafter, their colons were excised, washed with PBS, dissected longitudinally, fixed in 4% paraformaldehyde (PFA), embedded in paraffin and serially sectioned as described^9^. The Sect. (10-µm thick) were subjected to H&E staining. Images were taken with an Olympus FSX100 microscope and Olympus FSX100-BSW software. The severity of inflammatory colitis was also assessed by measuring the body weight loss and the colon length shortening.

### Intestinal carcinogenesis

Five weeks-old *Apc*^*Min/+*^ mice were administered intraperitoneally with ZPT, dipyrithione or the vehicle 3 days per week for 11 weeks. Thereafter, the mice were sacrificed and the whole small intestines were excised, rolled up in a ‘Swiss roll’ configuration, embedded in OCT compound and longitudinally sectioned at the 10-µm thickness. One section in the middle position of each intestine was subjected to H&E staining after fixation with 4% PFA, and tumours in the whole section were classified under microscope into hyperplasias, low-grade adenomas, high-grade adenomas and adenocarcinomas according to the histopathological criteria recommended for the study of mouse models of intestinal cancer^78^. The classification was conducted by a pathologist at Pathology Analysis Center (Kanagawa, Japan) under the blind condition regarding the compound administration.

### Tumour xenograft

Tumour xenograft experiments were performed as previously described^79^. Briefly, SW480 or Caco2 cells (5 × 10^6^ cells each) were implanted subcutaneously into the right flanks of female athymic nude (nu/nu) mice (6–8 weeks old, CLEA Japan). One week later, the mice were administered intraperitoneally with ZPT, dipyrithione or the vehicle 3 days per week for 3 or 8 weeks, respectively, during which the volumes (*V*) of the tumours, calculated by the following formula: *V* = *A* × *B*^2^/2 (*A*: the largest diameter, *B*: the perpendicular diameter), and the body weight were monitored every 7 days. Thereafter, tumours of the SW480-grafted mice were dissected, weighed, fixed in 4% paraformaldehyde, embedded in paraffin and serially sectioned. The Sect. (10-µm thick) were subjected to immunohistochemical detection of phosphorylated PKD by using anti-phospho-PKD antibody and HISTMOUSE-PLUS Kit (ZYMED Lab., Carlsbad, CA) according to the manufacturer’s protocol.

### Experimental lung metastasis

Female nude mice (6–8 weeks old) were injected with SW620 cells (1 × 10^7^ cells) suspended in 200 µl L-15 medium via tail vein and maintained for further 8 weeks. ZPT, dipyrithione or the vehicle was administered intraperitoneally to the mice twice, 1 week before and after the cell injection. Thereafter, the mice were sacrificed, and their lungs were excised, fixed in 4% PFA, embedded in paraffin and serially sectioned. The Sect. (10-µm thick) were subjected to H&E staining. For histologic evaluation of lung micro-metastasis, the number of micro-metastatic nodules in 3 non-contiguous lung sections from each mouse was counted under a light microscope as described previously^80^.

### Statistical analysis

Values are expressed as the means ± standard deviations (SD). Unpaired Student’s *t*-test was performed for determination of *p* values: *p < 0.05, **p < 0.01 and ***p < 0.005, using Graphpad Prism software (Graphpad Software Inc.). Differences were considered statistically significant in case the *p* values were smaller than 0.05.

## Supplementary Information

Below is the link to the electronic supplementary material.

Supplementary Material 1

## Data Availability

Data used and/or analyzed in the current study are available from the corresponding author upon request.
