# Supplementary material for: Increase of intracellular Zn2+ concentration directly inhibits phospholipase Cε and suppresses inflammation and tumour formation in mice
Source: Sci Rep. 2025 Nov 25;15:41853. doi: 10.1038/s41598-025-25886-5 (PMC12647759; doi:10.1038/s41598-025-25886-5)
Supplement: Supplementary file 1 — Supplementary Material 1 [file 41598_2025_25886_MOESM1_ESM.docx]

**Supplementary Table 1. Primers used for qRT-PCR analyses**

| Gene | Forward primer (from 5’ to 3’) | Reverse primer (from 5’ to 3’) |
| --- | --- | --- |
| CXCL1 | acccaaaccgaagtcatagc | tggggacaccttttagcatc |
| CXCL8 | cttggcagccttcctgattt | ttctttagcactccttggcaaaa |
| TNF-α | tgatcggtccccaaagg | ggtctgggccatagaactga |
| β-actin | atgaagatcaagatcattgctcctc | acatctgctggaaggtggacag |
| CCL2 | cacccagttctgctttggat | cgactgttgcctctcgtaca |
| CCL20 | ttgtcaccaagctcaagagaga | gaggtggttgtggaaaaggtag |
| COX-2 | gcaaattgctggcagggttg | agggcttcagcataaagcgt |


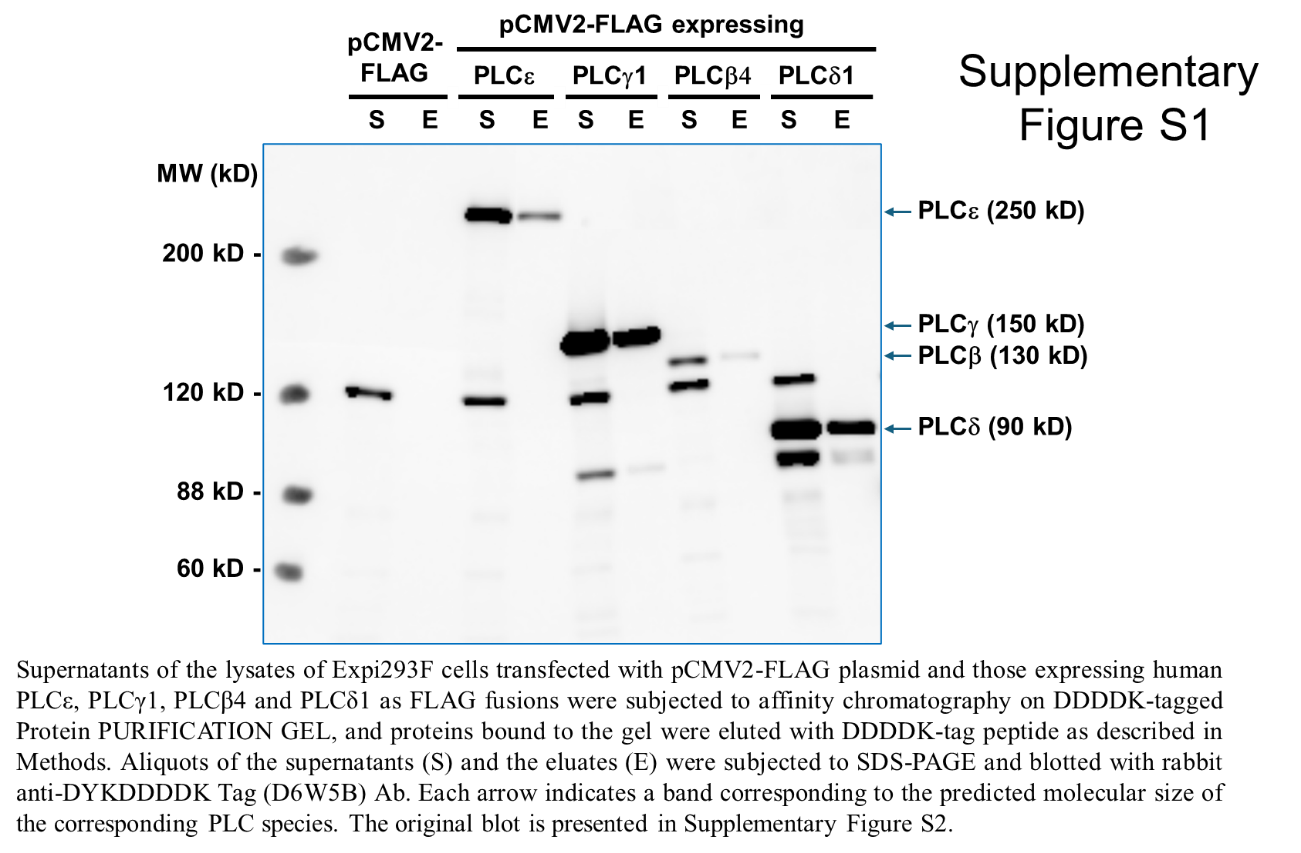


Supernatants of the lysates of Expi293F cells transfected with pCMV2-FLAG plasmid and those expressing human PLCε, PLCγ1, PLCβ4 and PLCδ1 as FLAG fusions were subjected to affinity chromatography on DDDDK-tagged Protein PURIFICATION GEL, and proteins bound to the gel were eluted with DDDDK-tag peptide as described in Methods. Aliquots of the supernatants (S) and the eluates (E) were subjected to SDS-PAGE and blotted with rabbit anti-DYKDDDDK Tag (D6W5B) Ab. Each arrow indicates a band corresponding to the predicted molecular size of the corresponding PLC species. The original blot is presented in Supplementary Figure S2.

Supplementary Figure S1


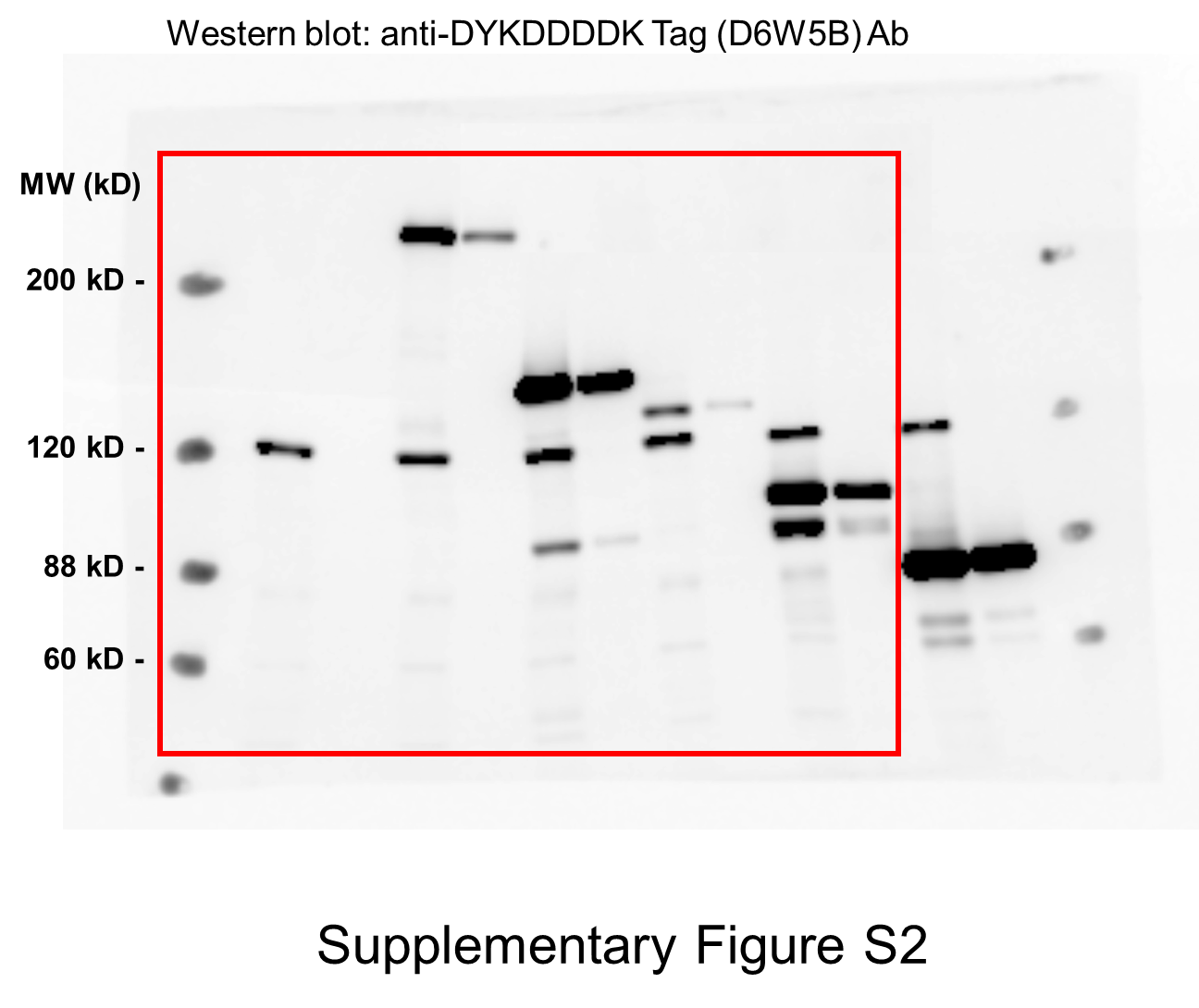


Supplementary Figure S2


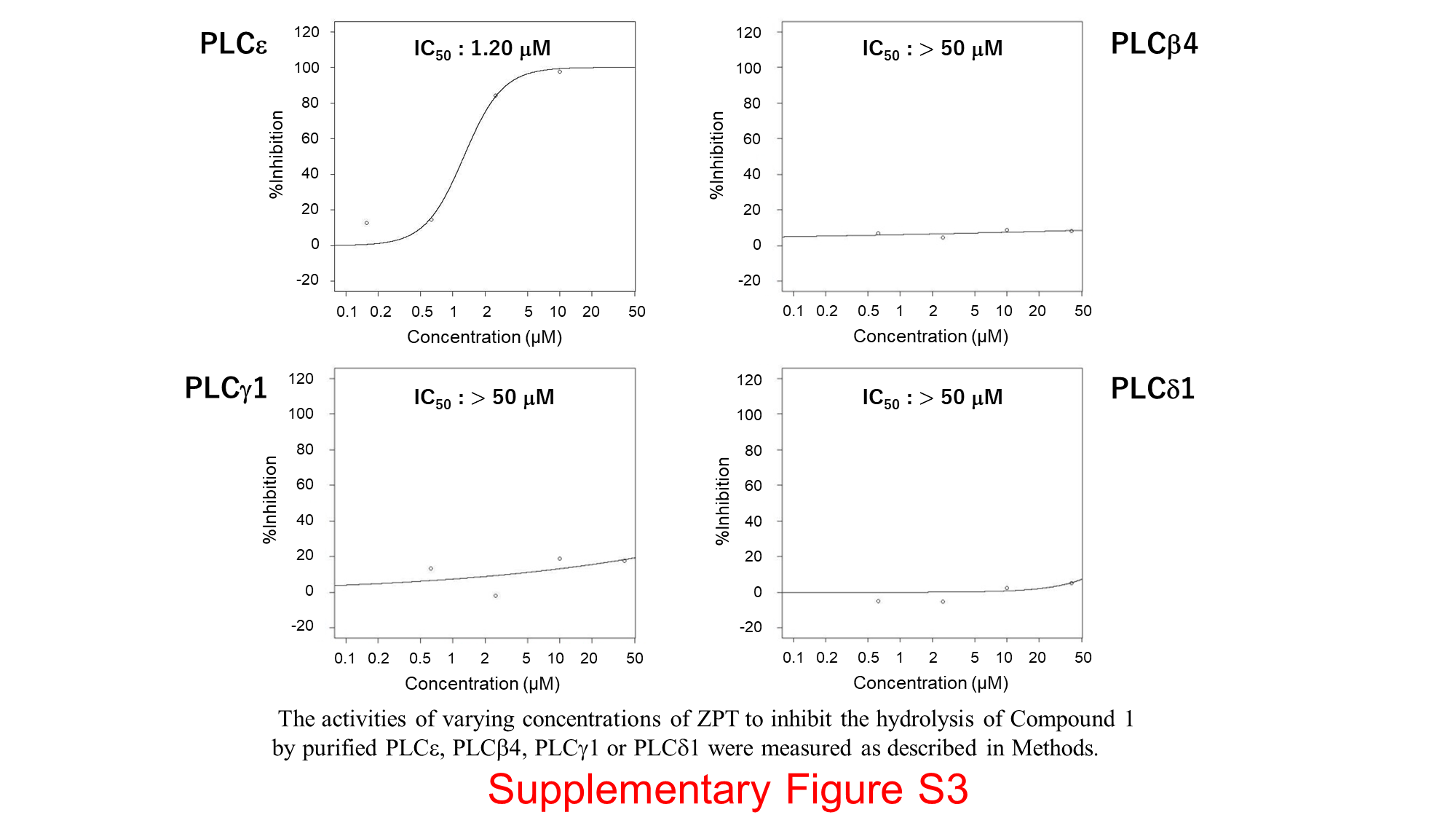


The activities of varying concentrations of ZPT to inhibit the hydrolysis of Compound 1 by purified PLCε, PLCβ4, PLCγ1 or PLCδ1 were measured as described in Methods.

Supplementary Figure S3


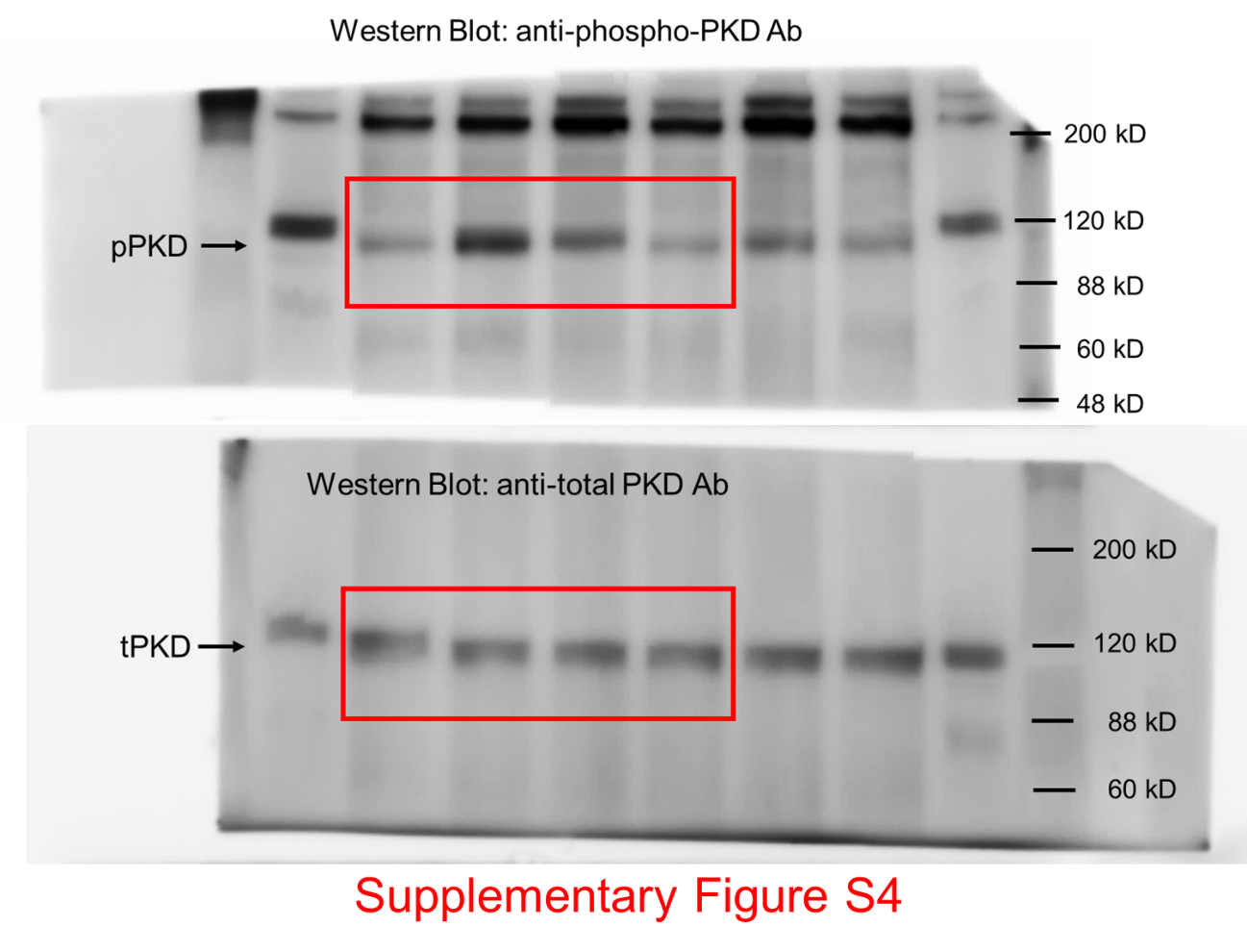


Supplementary Figure S4


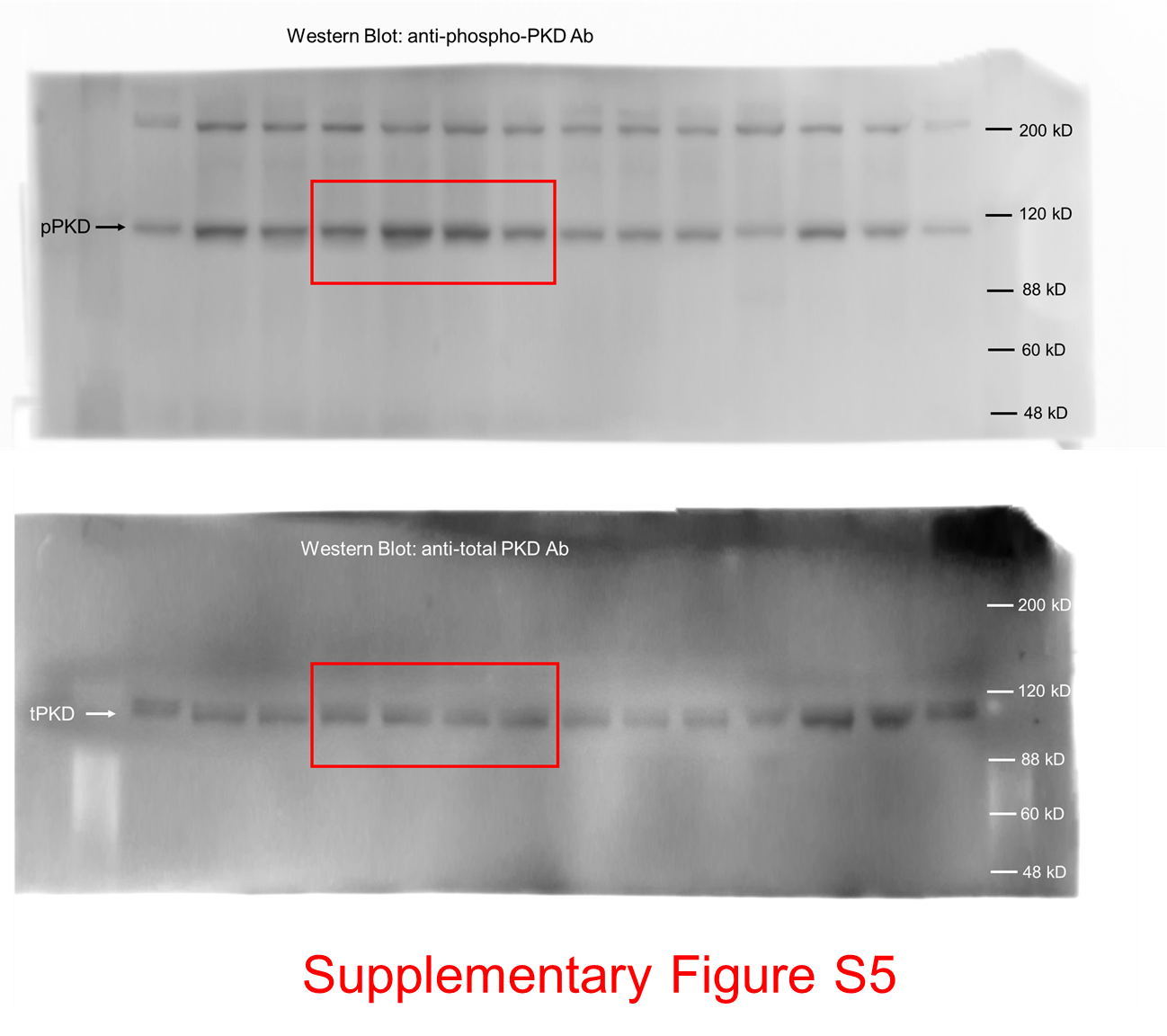


Supplementary Figure S5


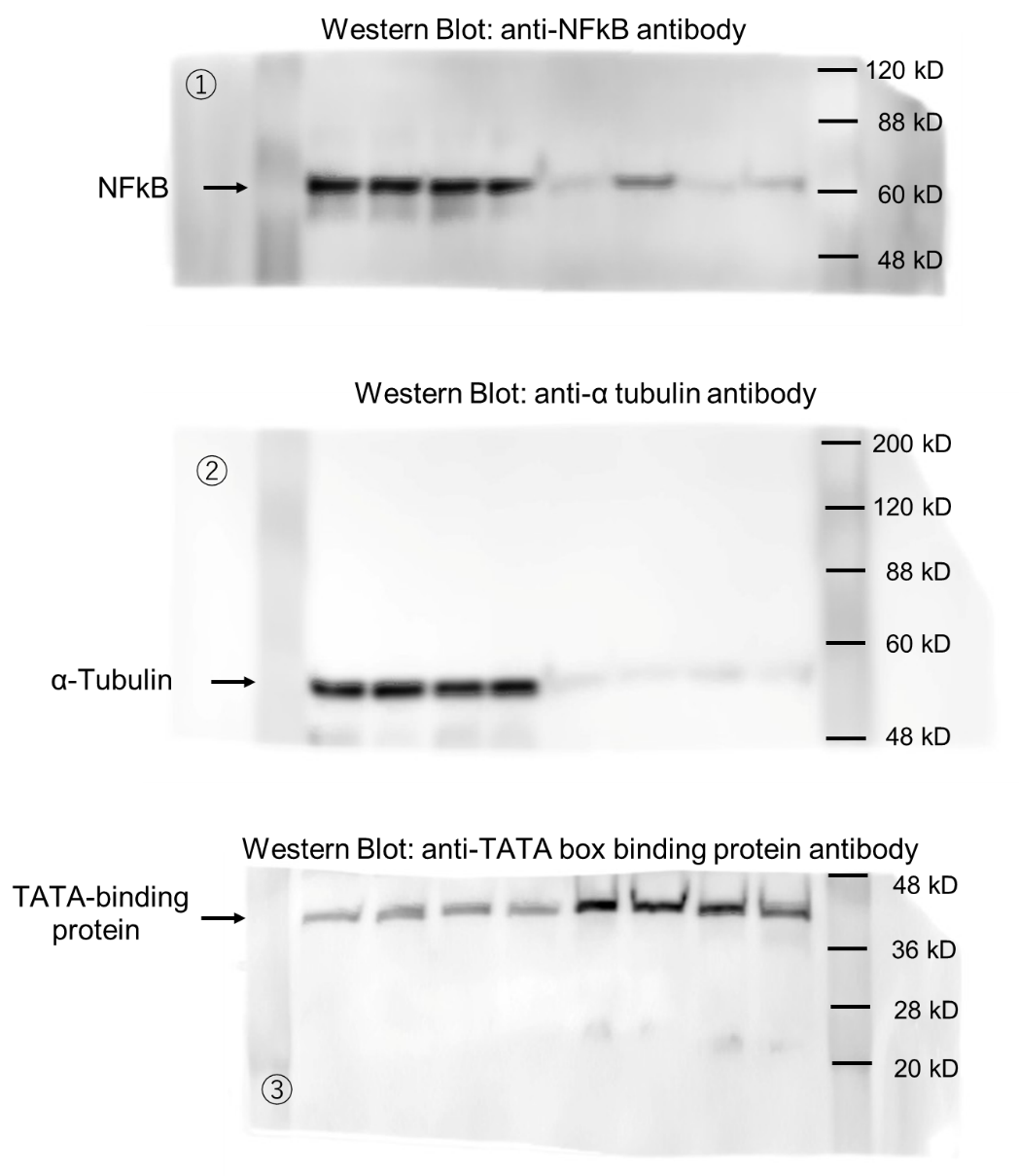


(② and ③) were derived from the same original blot, which was horizontally cut at the position corresponding to the 48-kD size marker.

Supplementary Figure S6


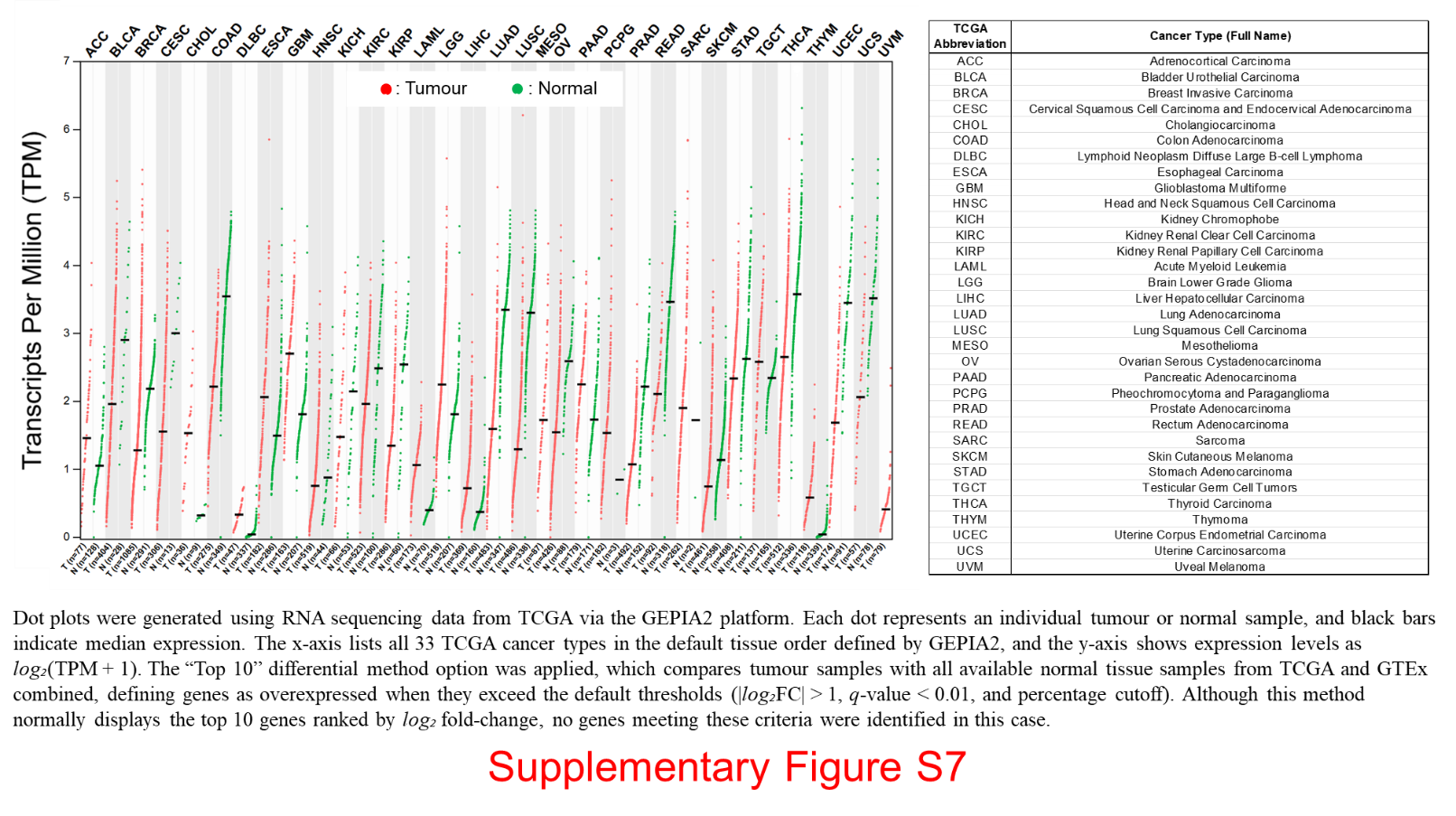


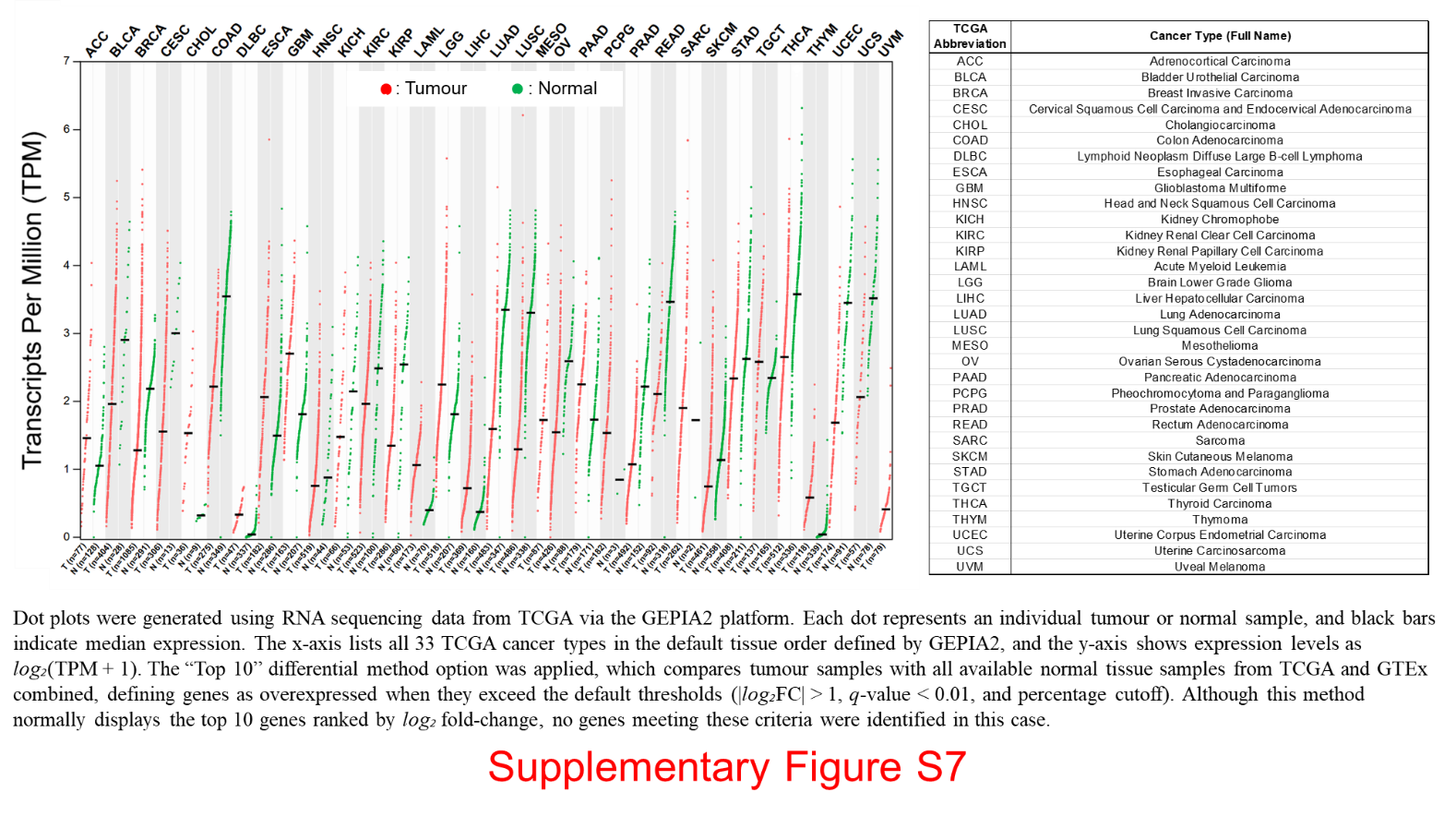


Dot plots were generated using RNA sequencing data from TCGA via the GEPIA2 platform. Each dot represents an individual tumour or normal sample, and black bars indicate median expression. The x-axis lists all 33 TCGA cancer types in the default tissue order defined by GEPIA2, and the y-axis shows expression levels as *log₂*(TPM + 1). The “Top 10” differential method option was applied, which compares tumour samples with all available normal tissue samples from TCGA and GTEx combined, defining genes as overexpressed when they exceed the default thresholds (|*log₂*FC| > 1, *q*-value < 0.01, and percentage cutoff). Although this method normally displays the top 10 genes ranked by *log₂* fold-change, no genes meeting these criteria were identified in this case.

Supplementary Figure S7


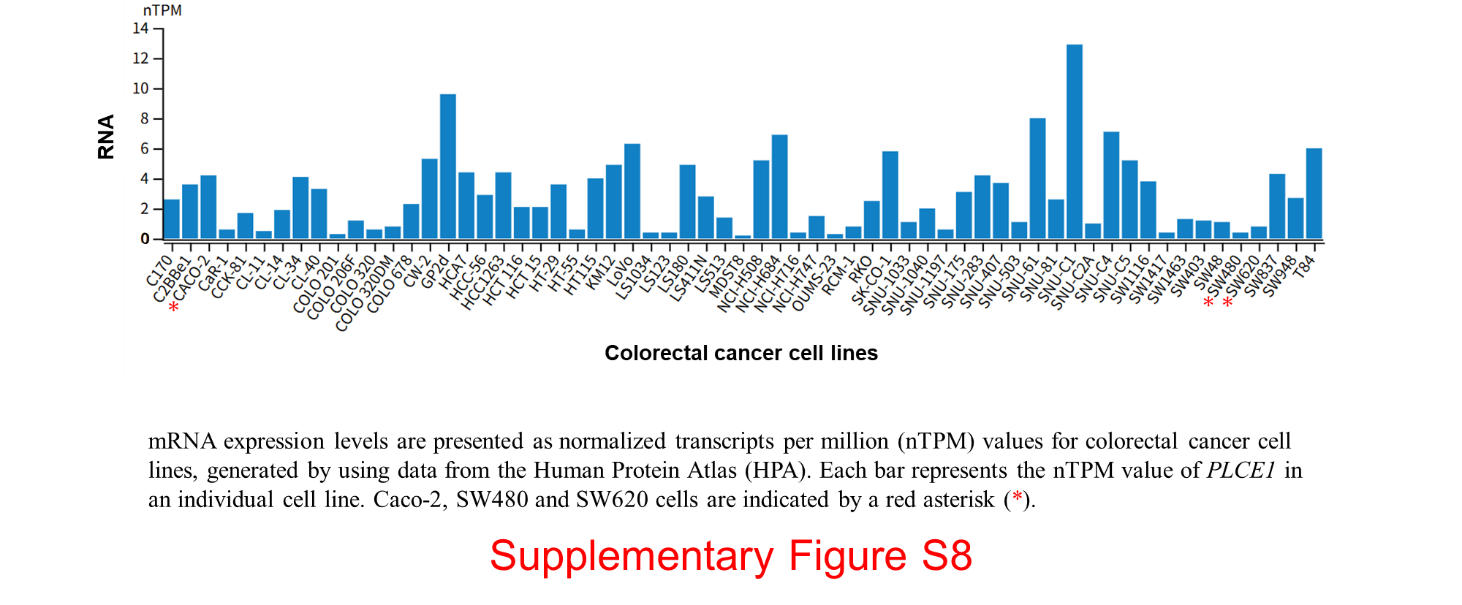


mRNA expression levels are presented as normalized transcripts per million (nTPM) values for colorectal cancer cell lines, generated by using data from the Human Protein Atlas (HPA). Each bar represents the nTPM value of *PLCE1* in an individual cell line. Caco-2, SW480 and SW620 cells are indicated by a red asterisk (*).

Supplementary Figure S8
